# Microbial Interactions Influence the Chemical Defense of Wild and Cultivated Tomato Species

**DOI:** 10.1007/s10886-025-01598-y

**Published:** 2025-04-07

**Authors:** Dimitri Orine, Haymanti Saha, Gaetan Glauser, Arjen Biere, Sergio Rasmann

**Affiliations:** 1 Institute of Biology, University of Neuchâtel, Rue Emile-Argand 11, Neuchâtel, Switzerland; 2 Department of Terrestrial Ecology, Netherlands Institute of Ecology (NIOO-KNAW), Droevendaalsesteeg 10, Wageningen, 6708 PB The Netherlands; 3 Neuchâtel Platform of Analytical Chemistry, University of Neuchâtel, Neuchâtel, 2000 Switzerland

**Keywords:** Biological control, Domestication, Glycoalkaloids, Metabolomics, Plant-microbe-insect interactions

## Abstract

Tomato, a globally significant crop, faces continuous threats from pests and pathogens, necessitating alternative approaches to reduce chemical inputs. Beneficial soil microbes, such as arbuscular mycorrhizal fungi (AMF) and plant growth-promoting rhizobacteria (PGPR), offer promising solutions by enhancing plant growth and pest tolerance. However, domestication may have weakened tomatoes’ interactions with these microbes, potentially compromising their innate immunity, a hypothesis that remains largely unexplored. To address this gap, we examined the effects of AMF and PGPR inoculation on growth, herbivory resistance, and metabolic responses in the domesticated *Solanum lycopersicum* ‘Moneymaker’ and three wild tomato relatives. Our findings reveal that microbial inoculation significantly influences both domesticated and wild tomatoes, with PGPR generally enhancing and AMF reducing plant growth across species. Using targeted and untargeted metabolomics, we found that soil microbes substantially alter plant chemistry above- and belowground in a species-specific manner. Notably, herbivore responses were more affected by AMF presence than by tomato species. These results highlight that while domestication has profoundly shaped tomato traits, microbial interactions can modulate these phenotypes. Thus, selecting microbial strains best suited to modern cultivars is crucial for optimizing plant growth and resilience against pests.

**Supplementary Information:**

The online version contains supplementary material available at 10.1007/s10886-025-01598-y.

## Introduction

One of the major challenges in nowadays’ agriculture is to maintain the necessary qualitative and quantitative food production, while imposing minimal harm to the environment (Lomba et al. 2020; Heyl et al. 2021). Among the different means for sustainable crop production, the use of root-associated microorganisms is being proposed as an environmentally-friendly way of increasing yield, as well as increasing plant resistance against pathogens and pests (Grover et al. 2011; Yu et al. 2019; Almario et al. 2022). For instance, arbuscular mycorrhizal fungi (AMF) have been shown to promote plant growth (Bennett and Bever 2007; Harman and Uphoff 2019), to augment plant defences against pathogens and insect herbivores (Papantoniou et al. 2021; Frattini et al. 2022), as well as to alleviate detrimental effects of abiotic stresses (Marro et al. 2022; Domingo et al. 2023). Similarly, inoculation with plant growth-promoting rhizobacteria (PGPR) has shown clear benefits for both plant development and defences (Vessey 2003; Meena et al. 2020). Yet, despite the widespread optimisms in using soil beneficial microbes for agriculture, several examples also show that microbe-plant-pest interactions are highly context dependent, varying tremendously across systems, and often failing to provide net benefits of using microbes for crop production (Kokkoris et al. 2019; Ray et al. 2020). Such variation can derive from different genotypes, both in plants and microbes, having different effects on each other (Hohmann and Messmer 2017), from the local environmental conditions, such as water, nutrients, and light availability (Saha et al. 2022; Orine et al. 2022; Wang et al. 2023; Nadeem et al. 2014), or from the local conditions in which the plant has developed (van der Putten et al. 2013; Revillini et al. 2016). Therefore, understanding the intrinsic sources of variation in plant-microbe insect interactions can spur new insights for optimising modern environmentally-friendly agriculture (Lee Díaz et al. 2021).

This problem is particularly accentuated when studying domesticated crops, since, during the domestication process, yield and food quality have always been favoured in spite of other traits, such as for example, plant-microbe associations (Fréville et al. 2022; Oyserman et al. 2022). Accordingly, recent evidence would indicate that wild species are better in exploiting the benefit of associating with soil microbes than their derived modern cultivars (Soldan et al. 2021). For example, in wheat, the degree of dependence on mycorrhizal symbiosis for plant growth and yield, was found to be lower in modern cultivars compared to ancestral genotypes and primitive landraces (Hetrick et al. 1993; Zhu et al. 2001). This reduced mycorrhizal dependence may be due to the highly fertile conditions used during plant breeding. However, a meta-analysis found that newer genotypes may be more mycorrhizal-responsive, but less intensively colonized (Lehmann et al. 2012). Similarly, domesticated cultivars of crops such as *Ensete ventricosum*, *Artocarpus* species, and *Zea mays* have been found to exhibit a lower capacity to support mycorrhizal symbiosis compared to their wild ancestors (Xing et al. 2012; Chu et al. 2013; Sangabriel-Conde et al. 2014; Garo et al. 2021). Consequently, it has been suggested that incorporating certain native landraces into cultivated fields could help maintain mycorrhizal symbiosis in agricultural ecosystems (Pérez-Jaramillo et al. 2016).

Tomatoes are one of the most widely grown vegetable crops worldwide, with a global production of more than 189 million tons in 2021 (2023). The commercial tomato species *Solanum lycopersicum* var. *lycopersicum* (Itkin et al. 2013), is the outcome of intense breeding programs from the pre-domesticated mid-wild tomato species *Solanum lycopersicum* var. *cerasiforme*, and nowadays diversified in a myriad of production cultivars, frequently more susceptible to insect pests and fungal pathogens than original wild tomato species (Steinkellner et al. 2012; Lebeda et al. 2014; Seifi et al. 2014). Concerning plant-soil microbe interactions, recent studies that compared different recent *S. lycopersicum* cultivars showed that susceptibility to AMF colonization can vary also between closely related cultivars (Gómez-Bellot et al. 2021), and that different tomato genotypes respond differently to a same bacterial endophyte (Poudel et al. 2019). Along these lines, it was shown that different cultivated and wild tomato genotypes have distinct but overlapping root bacterial microbiomes, and genetic variation in tomato can affect the global root bacterial microbiome (French et al. 2020). Nonetheless, very few studies have investigated and compared the effect of soil beneficial microbes on wild and domesticated tomato defences and resistance against herbivore pests.

Here, we explored variation in plant-microbe-insect pest interactions across wild relatives and modern tomato species. Specifically, our aim was to study chemical variation across tomato species and whether soil microbes can affect such variation, ultimately affecting plant resistance to pests. Indeed, both wild and cultivated plants produce a wide variety of specialized molecules, collectively known as the metabolome. This includes a range of secondary metabolites involved in withstanding biotic and abiotic stress (van Dam and Bouwmeester 2016; Turlings and Erb 2018). Particularly, tomato species are known to produce a range of alkaloids, and other specialized molecules to defend against herbivory. Tomatine and related compounds, for instance, are bitter-tasting alkaloids present in most parts of the plant that have been shown to protect tomato plants against pests and diseases (Dzakovich et al. 2020; Szymański et al. 2020; Akiyama et al. 2022). Yet, a range of other metabolites could also increase tomato resistance against insect pests, and in this regard, theory suggests that higher diversity of chemicals would increase resistance, either through complementary or synergistic effects (Messaili et al. 2021; Papantoniou et al. 2022; Roumani et al. 2022). Theory also proposes that the process of domestication would lead to a reduction in specialized metabolomic abundance diversity (Sonawane et al. 2018; Gao et al. 2019; Tohge et al. 2020), ultimately leading to lower resistance in cultivated crops compared to their wildtype relatives (Bolger et al. 2014; Ferrero et al. 2020; Kazachkova et al. 2021; Panda et al. 2021). However, how these chemical changes can be modified by soil beneficial microbes across tomato species remains to be fully elucidated.

By comparing three wild tomato species, and one commonly used modern tomato cultivar, we asked: (1) Is the increase in plant size and biomass production due to domestication also associated with a decline in chemical defences? (2) Is the domestication associated with a decrease in colonization rates of AMF in modern tomato plants? (3) Are AMF and PGPR generally beneficial for growth and defences across all tomato plant species? (4) Are AMF and PGPR inoculations changing the local and systemic (roots and leaves) metabolomes in a similar fashion across tomato species? We hypothesized that different tomato species differentially associate with beneficial microbes, and that the effect of beneficial microbes vary across species. Specifically, based on the domestication literature discussed above; we predicted that: (i) the commercial cultivar should grow bigger with improved yield; (ii) wild tomato species should be more likely to be colonized by AMF; (iii) wild species should be better in exploiting beneficial microbes than the domesticated species, associated with increased biomass and more defended plants; (iv) the root and leaf whole metabolome will be strongly influenced by the tomato species and microbial-induced effect on the metabolome might not be detectable in so different species.

## Methods and Materials

*Biological material* - To address our questions, we cultivated seedlings of four tomato species; the domestic species *Solanum lycopersicum* cultivar ‘Moneymaker’, obtained from Buzzy Seeds (Volendam, The Netherlands), and three wild tomato species (*Solanum arcanum* (CGN15801); *Solanum neorickii* (CGN24193); *Solanum pennelllii* (CGN15533)), all obtained from the Centre for Genetic Resources (The Netherlands), and regenerated at the Botanical Garden of Neuchâtel (Switzerland) under standard greenhouse conditions with natural summer light length and temperature. The wild species originated from Peru and Ecuador, each with distinct bioclimatic preferences, such that *S. arcanum* is tendentially found on the western slopes of the Andes, *S. neorickii* on higher elevation mountain sites, and *S. pennellii* being a predominantly coastal species (Supplementary material, Fig. S1). The arbuscular mycorrhizal fungus (AMF) *Rhizoglomus irregularis* MUCL-57021, was acquired from Koppert B.V. (Berkel en Rodenrijs, The Netherlands) as spore suspension in sterilized water. The plant growth-promoting rhizobacteria (PGPR) *Pseudomonas protegens* Strain CHA0 (phylum: *Pseudomonadota)* (formerly *Pseudomonas fluorescens*, phylum: *Proteobacteria*) was cultivated for 48 h in liquid nutrient medium (22 °C, 60 rpm) from a pre-culture originated from the laboratory of Microbiology at the University of Neuchâtel (Switzerland).

*Experimental desig*n - We performed a full factorial experimental design with 4 tomato species (*S. lycopersicum* (L), *S. arcanum* (A), *S. neorickii* (N), *S. pennelllii* (P)) and 3 different microbial treatments (Control (C); Bacteria: *P. protegens* (P); AMF: *R. irregularis* (R)), with 20 replicates per treatment combination (*N* = 240 plants in total). The experiment took place in the glasshouse facility at the Netherlands Institute of Ecology (NIOO-KNAW), Wageningen, The Netherlands. For germination, tomato seeds were first surface-sterilized in a water-sodium hypochlorite (5% NaClO) solution for 10 min, rinsed thoroughly with deionized water and then germinated on gamma-sterilized potting soil in cell seedling trays (4 cm deep holes). The seedlings were maintained in the greenhouse with natural daylight at 24 °C/18°C day/night until transplantation 24 days after germination. For each treatment group, 20 individual seedlings (*n* = 20) were transferred to 1 L plastic pots containing a sterile mixture of potting soil and quartz sand (3:1, v: v). To avoid microbial mixing between treatments, each pot was placed on a saucer. At transplantation, seedlings were inoculated with microbes by adding a 10 mL liquid solution to the soil directly at the root zone: plants of the AMF treatment received a 50 spores per mL inoculum (*R. irregularis* (R)) in sterile water. Plants of the bacterial treatment received a culture (OD_600nm_ = 0.8) inoculum (*P. protegens* (P)). The control plants (C) and AMF treated plants (R) received 10 mL of sterile culture medium, in order to homogenize the inoculation media. Plants were watered until field capacity with tap water and fertilized twice a week with half-concentration Hoagland nutrient solution from 50 mL at first week to 150 mL at the end of culture. Growing conditions consisted in constant temperature (22 ± 2 °C) and 70% relative humidity with a daylight photoperiod of 16 h. Additional lighting was provided between 06:00 h and 22:00 h by high pressure sodium lamps (Son-T, 600 W Philips GreenPower; Koninklijke Philips N.V., Amsterdam, The Netherlands) that were automatically switched on when light intensity dropped below 225 µmol m^− 2^ s^− 1^.

*Measurements of functional traits and sampling* - During the experiment, the length of the plants was weekly measured from the cotyledon insertion point to the terminal apex, from week one to week six. At the end of the experiment, eight weeks after transplanting, the following functional traits related to plant growth and biomass accumulation were measured on all replicates: plant height (mm), measured as the main stem length, width of main stem at the cotyledon insertion point (mm), aboveground fresh biomass (leaves and stems), belowground fresh biomass (roots), and fruit fresh biomass. A fraction of the fresh roots was collected and stored in ethanol for AMF colonization rate determination, and finally, fresh leaf and root samples were collected and fast frozen in liquid nitrogen for metabolomic analyses after lyophilisation (see below).

*Insect resistance bioassay* - The insect bioassay experiment was performed seven weeks after inoculation on a subset of the experimental plants (*n* = 5). For the herbivory bioassay we used the tomato looper *Chrysodeixis chalcites* (*Lepidoptera: Noctuidae*), originally obtained from Wageningen University and Research Center in Bleiswijk (NL), and maintained at the Netherlands Institute of Ecology, Wageningen (NL). The caterpillars were reared on *Solanum lycopersicum* tomato leaves at 22 °C, 16 h photoperiod and 50% relative humidity until reaching instar L3. Five replicates per treatment combination were used for this bioassay on three tomato species combined to the three microbial treatments, for a total of 135 caterpillars. For each replicate, three third-instar caterpillars were weighed together on a microbalance (Mettler Toledo Instruments, Columbus, Ohio, United States) and released in a 14 cm petri dish with the youngest fully expanded leaf. The leaf blade with all leaflets was fitted inside the Petri dish sealed with parafilm, while the petiole was sticking out from a hole, plugged with cotton and inserted inside a 5 ml Eppendorf filled with water. This setup allowed the detached leaf to stay fresh for the duration of the bioassay. All Petri dishes were randomly placed and monitored for 72 h at 22 °C and 50% RH inside a growth chamber. At the end, as all the three caterpillars survived in all bioassays, they were weighed again together, and their weight averaged by plant. For each plant replicate, the weight gain ratio of the caterpillars was finally calculated as *(final weight - initial weight) / initial weight*. Insect performance could not be tested on *S. pennellii* for which leaves are covered with trichomes releasing important amount of sticky acylsugars repelling caterpillars.

*Determination of mycorrhizal inoculation* - Mycorrhizal inoculation was determined after carefully cleaning the roots under tap water. The roots were washed in KOH (10%) for 30 min at 90 °C and rinsed with water acidified with acetic acid (1%). The fungal structures present in the roots were stained with a staining solution (5% blue ink dissolved in 5% acetic acid) following the method described in (Vierheilig et al. 2005). Mycorrhizal colonization was assessed using the gridline intersection method (Giovannetti and Mosse 1980) under a stereo microscope at 100x magnification (Leica M205C). We calculated the colonization rate as the percentage of root length colonized by the AMF, considering hyphae, arbuscules and vesicles together.

*Plant secondary metabolites analyses* - Both an untargeted (using data-independent mode, DIA) and a targeted (focusing on the quantification of tomato glycoalkaloids) metabolomics approach were used to assess the effect of microbial inoculum on metabolites across the four different tomato species. Complete details on extraction, sample analysis and data processing are available in Methods S1-S3.

*Statistical analysis -* Analyses were conducted using the software R4.0.5 (R Core Team 2023).

To assess the effect of the microbial inoculation treatments (two levels), tomato species (four levels), and their interaction effect on plant growth and defences, the growth traits (plant height, belowground biomass, aboveground biomass, stem width, fruit fresh weight), the mycorrhizal colonization and the insect weight gain were compared across all treatment combinations using two-way univariate analyses of variance (ANOVAs). ANOVA assumptions were checked on residuals. When needed the variables were log-transformed before analyses to comply with the requirement of homoscedasticity of variance. Similarly, the interactive effect of microbial inoculations and species on the log-transformed concentration of targeted glycoalkaloids (alpha-tomatine, dehydrotomatine, hydroxytomatine, acetoxytomatine (I), acetoxytomatine (II), dehydro-acetoxytomatine) in roots and leaves were assessed with two-way ANOVAs, separately for both organs. To summarize chemical defence of the different tomato species on a bi-dimensional space, all targeted glycoalkaloids measured in the leaves were ordinated using a principal component analysis (PCA) calculated with the ‘ade4’ package (Dray and Dufour 2007). To analyse the untargeted leaf and root metabolome we used the ‘mixOmics’ package (Rohart et al. 2017a; b). A sparse Partial Least Squares Discriminant Analysis (sPLS-DA) using Multivariate INTegrative method (MINT) was conducted to assess the potential to discriminate the samples according to the tomato species or the microbial treatment and identify chemical features signatures characterizing species or microbe effect (Hervé et al. 2018). Complete details of the statistical analyses led on the the ‘mixOmics’ package are available in Methods S4.

## Results

**Fig. 1:**
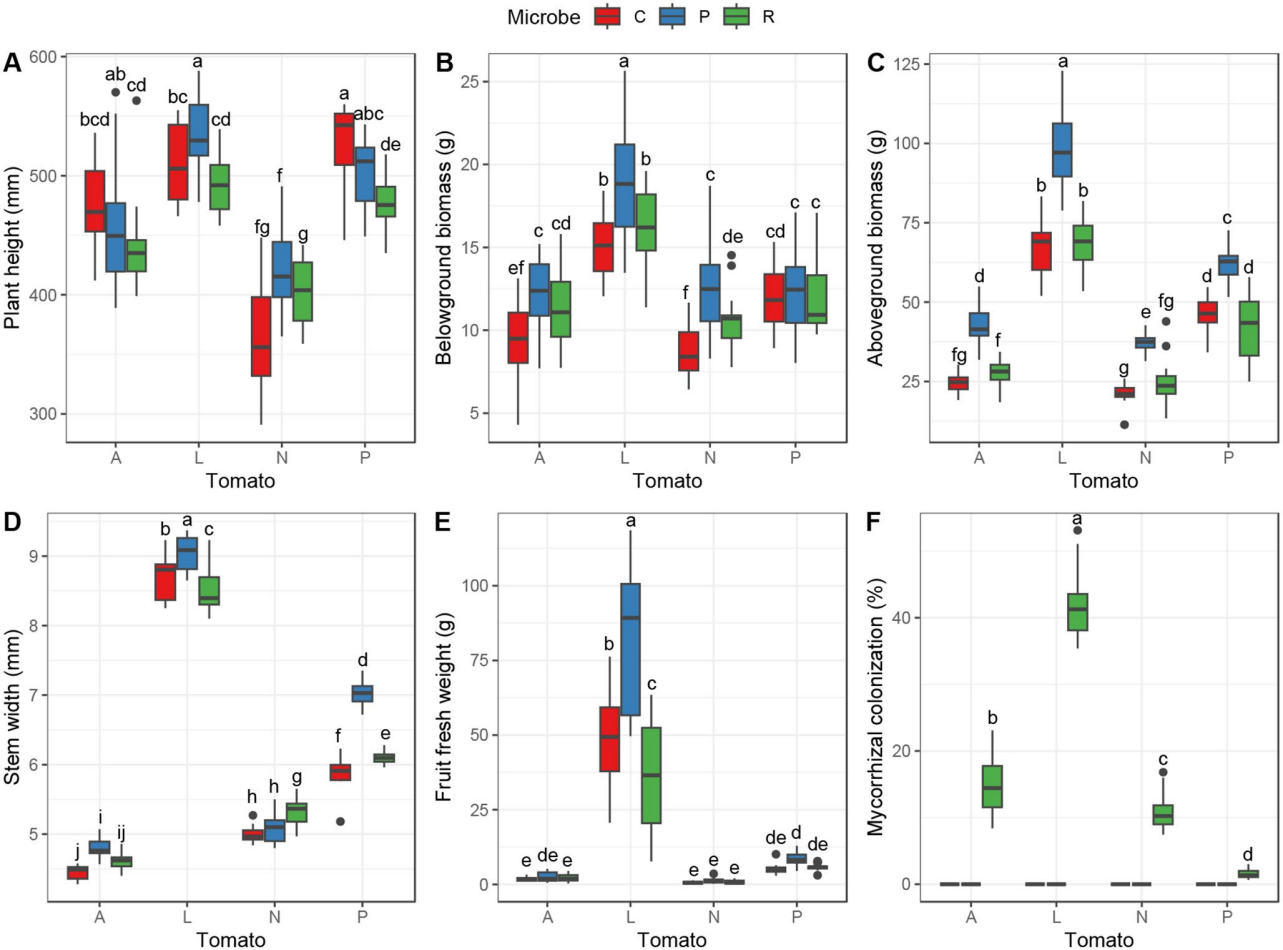
Tomato species and microbe effects on plant growth. Boxplots show for tomato species (A: *S. arcanum*; L: S. *lycopersicum*; N: *S. neorickii*; P: *S. pennellii*) and each microbial treatment (C: control; P: *Pseudomonas protegens*; R: *Rhizophagus irregularis*) the raw data of the recorded variables: plant height at six weeks after transplantation (**A**); and belowground biomass (**B**) aboveground biomass; (**C**); stem width (**D**); fruits fresh weight (**E**); Mycorrhizal colonization (**F**), all measured at eight weeks after transplantation. Letters above boxplots indicate significant differences among tomato species and microbe treatments (Tukey HSD test, *p* < 0.05). Boxplots represent, from bottom to top, minimum, first quartile, median, third quartile and maximum, and dots represent the outliners

*Plant growth traits and AMF colonization across tomato species* - All plant-growth traits measured varied across the tomato species tested (Table 1). Specifically, while the domesticated species *S. lycopersicum* ‘moneymaker’ had similar height as *S. pennellii*, around 280 mm at 4 weeks after inoculation and 500 mm at 6 weeks, it produced the highest belowground and aboveground biomasses compared to all the three wild species (Fig. 1A, B and C). The plants from the species *S. neorickii* were the smallest, 22.5% less than *S. lycopersicum*, for all microbial treatments averaged (Fig. 1A). When comparing within the control treatment, *S. arcanum* and *S. neorickii* both produced the lowest root and shoot biomasses. Specifically, *S. pennellii* had 12% higher root biomass and 58% higher shoot biomass than *S. arcanum.* Stem width followed the same trend, as *S. arcanum* had the narrowest stem width of 4.5 mm, *S. neorickii* 5.2 mm, *S. pennellii* 6.5 mm, while the domesticated cultivar produced the widest stems, between 8 and 9 mm in average (Fig. 1D). Confirming the domestication tendency, fruit weight was largely higher for *S. lycopersicum* with an average of 55 g per plant, despite plants were still young, while *S. pennellii* fruits weighed less than 7 g on average and even less for the other wild species (Fig. 1E). Finally, we observed that the roots of the domesticated variety had on average 41.6% AMF colonization (Fig. 1F), while the wild species all had less than 20% AMF colonization, with *S. arcanum* roots being colonized at 14.7%, *S. neorickii* at 10.8%, and *S. pennellii* at 1.5% (Fig. 1F; Table 1).

**Table 1:** Two-way interaction ANOVA table for measuring the effect of microbe treatments (three levels) and tomato species (four levels) on plant growth (plant size at six weeks after transplanting), aboveground fresh weight biomass (AG), belowground fresh weight biomass (BG), stem width, fruits fresh weight, arbuscular mycorrhizal fungi (AMF) colonization (all at eight weeks after transplanting), and *Chrysodeixis chalcites* caterpillars weight gain (Caterpillar) at seven weeks after transplanting. Log means that data were log10-transformed prior analysis

Plant traits	Treatment	Df	SumSq	F value	*p*-value
Plant size	Microbe (M)	2	16,345	5.92	0.003
	Tomato species (S)	3	295,801	71.37	< 0.001
	M x S	6	35,109	4.24	< 0.001
	Residuals	132	182,376		
AG (log)	M	2	9.87	210.791	< 0.001
	S	3	41.88	596.177	< 0.001
	M x S	6	0.61	4.348	< 0.001
	Residuals	228	5.34		
BG	M	2	313	28.215	< 0.001
	S	3	1494	89.792	< 0.001
	M x S	6	110.2	3.311	0.004
	Residuals	228	1264.5		
Stem width (log)	M	2	0.146	66.26	< 0.001
	S	3	8.565	2588.58	< 0.001
	M x S	6	0.146	22.05	< 0.001
	Residuals	132	0.146		
Fruits (log)	M	2	12.7	19.051	< 0.001
	S	3	628.3	630.415	< 0.001
	M x S	6	5.2	2.591	0.019
	Residuals	228	75.7		
AMF (log)	M	3	28.5	580.9	< 0.001
	S	2	320.5	9796.8	< 0.001
	M x S	6	57	580.9	< 0.001
	Residuals	228	3.7		
Caterpillar (log)	M	2	0.2845	3.869	0.030
	S	2	0.2298	3.126	0.056
	M x S	4	0.1216	0.827	0.517
	Residuals	36	1.3233		

*Soil microbial effects on plant growth traits across tomato species* - Overall, the microbial treatments significantly affected the plant growth and had different effects depending on the tomato species (Fig. 1) as expressed by the significant interaction effect between tomato species and microbial treatment for all traits measured (Table 1). For plant height, plants inoculated with *P. protegens* were always taller than control, but this effect was only significant for the domesticated species *S. lycopersicum* ‘moneymaker’ and for *S. neorickii* (Fig. 1A). Specifically, growth curves show that the microbial treatments started to influence plant size from the third week after inoculation (Fig. S2). Moreover, five weeks after inoculation, all the wild species started to produce lateral stems with random architecture, thus generating more variability in plant height for the cultivated species (Fig. S2).

Belowground biomass was higher for *P. protegens* inoculated plants except for the species *S. pennellii*, which showed no difference between microbial treatments and the control (Fig. 1B). The PGPR increased root biomass by 30.8%, 27.5% and 44.7%, for *S. arcanum*, *S. lycopersicum* and *S. neorickii*, respectively. AMF-inoculated plants also had significantly higher root biomass than the control for the species *S. arcanum* and *S. neorickii*, with an increase of 24.3% and 20.9% respectively.

Aboveground biomass was similar between the control treatment and the *R. irregularis*-inoculated plants, while *P. protegens* inoculated plants were heavier for all plant species (Fig. 1C). Moreover, compared to the PGPR treatments, the leaves and stems biomass of the AMF treatments were lighter by 35.0% for *S. arcanum*, 29.4% for *S. lycopersicum*, 33.1% for *S. neorickii*, and 31.9% for *S. pennellii*.

Stem width was also impacted by the microbial treatment (Fig. 1D). Particularly, the PGPR treatment induced 6.1% thicker stems for *S. lycopersicum*, and 14.9% thicker stem for *S. pennellii* than the AMF treatment, respectively.

Finally, the fruit fresh weight of *S. lycopersicum* ‘moneymaker’ plants inoculated with *P. protegens* was 69% higher than the control treatment, while the same species inoculated with *R. irregularis* had a 25% lower yield than the control. For the wild species, we could not detect a microbial treatment effect on fruit yield (Fig. 1E).

**Fig. 2:**
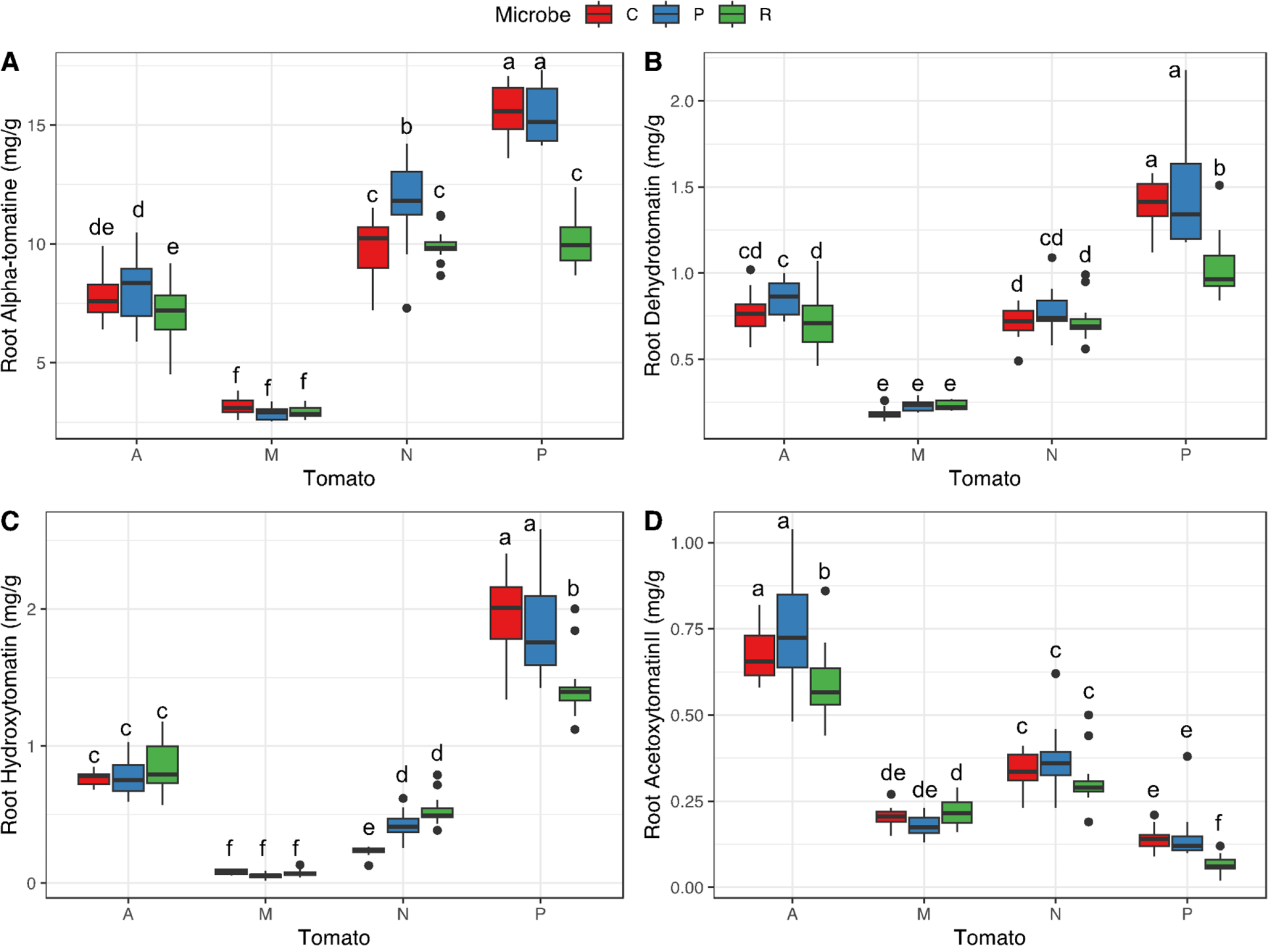
Tomato species and microbe effects on targeted chemical concentration in the roots. Boxplots show for each tomato species (**A**: *S. arcanum*; L: *S*. *lycopersicum*; N: *S. neorickii*; P: *S. pennellii*) and microbial treatments (C: control; P: *Pseudomonas protegens*; R: *Rhizophagus irregularis*) the raw data of the recorded variables: alpha-tomatine (**A**); dehydrotomatine (**B**); hydroxytomatine(**C**); acetoxytomatine II (**D**). Letters above boxplots indicate significant differences among tomato species and microbe treatments (Tukey HSD test, *p* < 0.05). Boxplots represent, from bottom to top, minimum, first quartile, median, third quartile and maximum, and dots represent the outliners

**Fig. 3:**
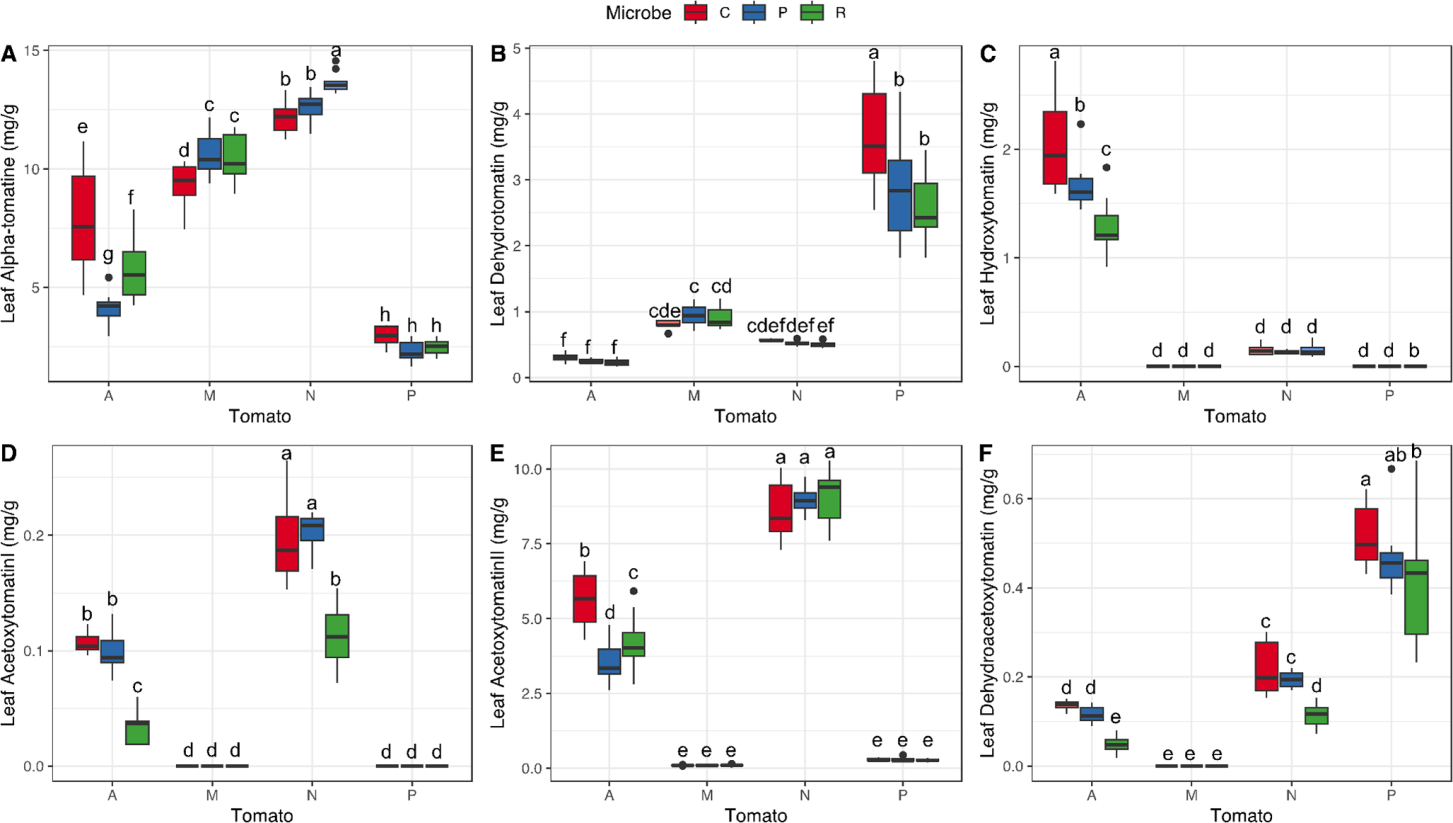
Tomato species and microbe effects on targeted chemical concentration in the leaves. Boxplots show for tomato species (A: *S. arcanum*; L: S. *lycopersicum*; N: *S. neorickii*; P: *S. pennellii*) and microbial treatment (C: control; P: *Pseudomonas protegens*; R: *Rhizophagus irregularis*) the raw data of the recorded variables: alpha-tomatine (**A**); dehydrotomatine (**B**); hydroxytomatine (**C**); acetoxytomatine I (**D**); acetoxytomatine II (**E**); dehydroacetoxytomatine (**F**). Letters above boxplots indicate significant differences among tomato species and microbe treatments (Tukey HSD test, *p* < 0.05). Boxplots represent, from bottom to top, minimum, first quartile, median, third quartile and maximum, and dots represent the outliners

*Glycoalkaloids’ variation across tomato species–* Leaf glycoalkaloids varied strongly across species (Table 2), with some compounds generally being produced together, while others being specific to some species (Fig. S3). In roots, four glycoalkaloids were present in quantifiable amount and had significantly different concentrations depending on the tomato species (Table 2). Alpha-tomatine was the compound with the highest concentration in the root for all four species, with highest amounts above 15 mg/g of dry root in *S. pennellii*, and the lowest amounts in *S. lycopersicum* ‘moneymaker’ with 3 mg/g (Fig. 2A). Compared to the three wild species, *S. lycopersicum* also had the lowest concentrations of dehydrotomatine and hydroxytomatine, while *S. pennellii* had the highest concentration of these two molecules, with around 1.5 and 2 mg/g respectively (Fig. 2B, C). Acetoxytomatine (II) was the glycoalkaloid with the lowest concentrations in the roots, with 0.5 to 1 mg/g in *S. arcanum* and the minimum in *S. pennellii* with only 0.1 mg/g (Fig. 2D). In leaves, two other compounds (acetoxytomatine (I) and dehydroacetoxytomatine) were present in sufficient amounts to allow quantification. For all the six glycoalkaloids, we found a significant effect of the tomato species on the molecule’s concentrations (Table 2), but effects for each molecule were idiosyncratic across species (Fig. 3). For alpha-tomatine, the highest concentrations were found in *S. neorickii* (between 12 and 15 mg of compound per g of dry leaf), respectively followed by *S. lycopersicum* ‘moneymaker’ and *S. arcanum* (Fig. 3A). *S. pennellii* showed the lowest concentration in alpha-tomatine. Contrarily, *S. pennellii* was the species producing the highest amounts of dehydrotomatine (2 to 5 mg/g). Concerning hydroxytomatine, both *S. lycopersicum* and *S. pennellii* showed concentrations below the quantification limit of 0.1 mg/g, *S. neorickii* procured barely detectable amounts, while *S. arcanum* produced between 1 mg and 3 mg of this compound per g of leaf. Interestingly, acetoxytomatine (II) was the second compound with highest concentrations in the leaves of both *S. neorickii* and *S. arcanum*, while *S. lycopersicum* and *S. pennelli* did not produce this compound in detectable concentrations. Acetoxytomatine (I) followed a similar trend, but with much lower concentrations. Dehydroacetoxytomatine was present in the leaves of wild tomato species with concentrations below 1 mg/g, while completely absent from the domesticated species *S. lycopersicum* ‘Moneymaker’, since we found that this modern cultivar only produced two glycoalkaloids, alpha-tomatine and dehydrotomatine, in quantifiable amounts in the leaves (Fig. 3).

**Table 2:** Two-way interaction ANOVA table for measuring the effect of microbe treatments (three levels) and tomato species (four levels) on leaf and root alkaloids (alpha-tomatine, Dehydrotomatine, acetoxytomatine (II), hydroxytomatine, dehydroacetoxytomatine, acetoxytomatine (I))

Organ	Chemical compound	Treatment	Df	SumSq	F value	*p*-value
Leaf	alpha-tomatine	Microbe (M)	2	0.62	12.155	< 0.001
		Tomato species (S)	3	38.39	498.778	< 0.001
		M x S	6	1.35	8.782	< 0.001
		Residuals	86	2.21		
	Dehydrotomatine	M	2	0.37	5.386	0.006
		S	3	76.63	747.556	< 0.001
		M x S	6	0.56	2.716	0.018
		Residuals	86	2.94		
	Acetoxytomatine (II)	M	2	0.5	7.857	< 0.001
		S	3	345.7	3949.754	< 0.001
		M x S	6	0.9	4.919	< 0.001
		Residuals	86	2.5		
	Hydroxytomatine	M	2	0.082	10.17	< 0.001
		S	3	15.821	1304.82	< 0.001
		M x S	6	0.25	10.31	< 0.001
		Residuals	86	0.348		
	Dehydroacetoxytomatine	M	2	0.0649	20.463	< 0.001
		S	3	1.8816	395.566	< 0.001
		M x S	6	0.0224	2.352	0.037
		Residuals	86	0.1364		
	Acetoxytomatine (I)	M	2	0.0276	68.49	< 0.001
		S	3	0.4153	685.88	< 0.001
		M x S	6	0.0289	23.86	< 0.001
		Residuals	86	0.0174		
Root	alpha-tomatine	M	2	0.74	29.14	< 0.001
		S	3	34.01	891.89	< 0.001
		M x S	6	0.82	10.8	< 0.001
		Residuals	132	1.68		
	Dehydrotomatine	M	2	0.57	11	< 0.001
		S	3	62.67	801.24	< 0.001
		M x S	6	0.97	6.23	< 0.001
		Residuals	132	3.44		
	Acetoxytomatine (II)	M	2	0.048	7.914	< 0.001
		S	3	3.255	357.448	< 0.001
		M x S	6	0.058	3.192	0.006
		Residuals	132	0.401		
	Hydroxytomatine	M	2	0.008	0.74	0.479
		S	3	17.126	1079.1	< 0.001
		M x S	6	0.546	17.2	< 0.001
		Residuals	132	0.698		

*Microbial inoculum effects on glycoalkaloids -* The concentration of all quantifiable root glycoalkaloids varied depending on the microbial treatment, except for hydroxytomatine (Fig. 2; Table 2). Overall, while the PGPR did not affect root alkaloids’ concentrations, inoculation with the AMF tended to decrease them (Fig. 2). More specifically, the biggest impact of microbes was found for *S. pennellii*, in which *R. irregularis* decreased the concentrations of alpha-tomatine by 35%, dehydrotomatine by 26.8% and hydroxytomatine by 26.8%, compared to the control (Fig. 2).

Similarly to roots, the concentration of the six glycoalkaloids found in leaves was affected by the microbial treatment (Fig. 3; Table 2), including species by microbe interaction (Table 2). Alpha-tomatine increased in *S. neorickii* by 11.9% when inoculated with AMF, while the same compound decreased in *S. arcanum* by 24% and by 46% when inoculated with AMF and with PGPR, respectively, compared to control plants. For all other compounds, when significant differences were there, microbial treatments generally decreased their concentrations in leaves compared to control plants. For dehydrotomatine, in *S. pennellii*, concentrations were 20% lower for PGPR-inoculated plants and 29% lower for AMF-inoculated ones. For hydroxytomatine, in *S. arcanum*, concentrations were 19% lower for PGPR-inoculated plants and 37% lower for AMF-inoculated ones. For acetoxytomatine (II) in *S. arcanum*, concentrations were 27% lower for PGPR-inoculated plants and 25% lower for AMF-inoculated ones.

**Fig. 4:**
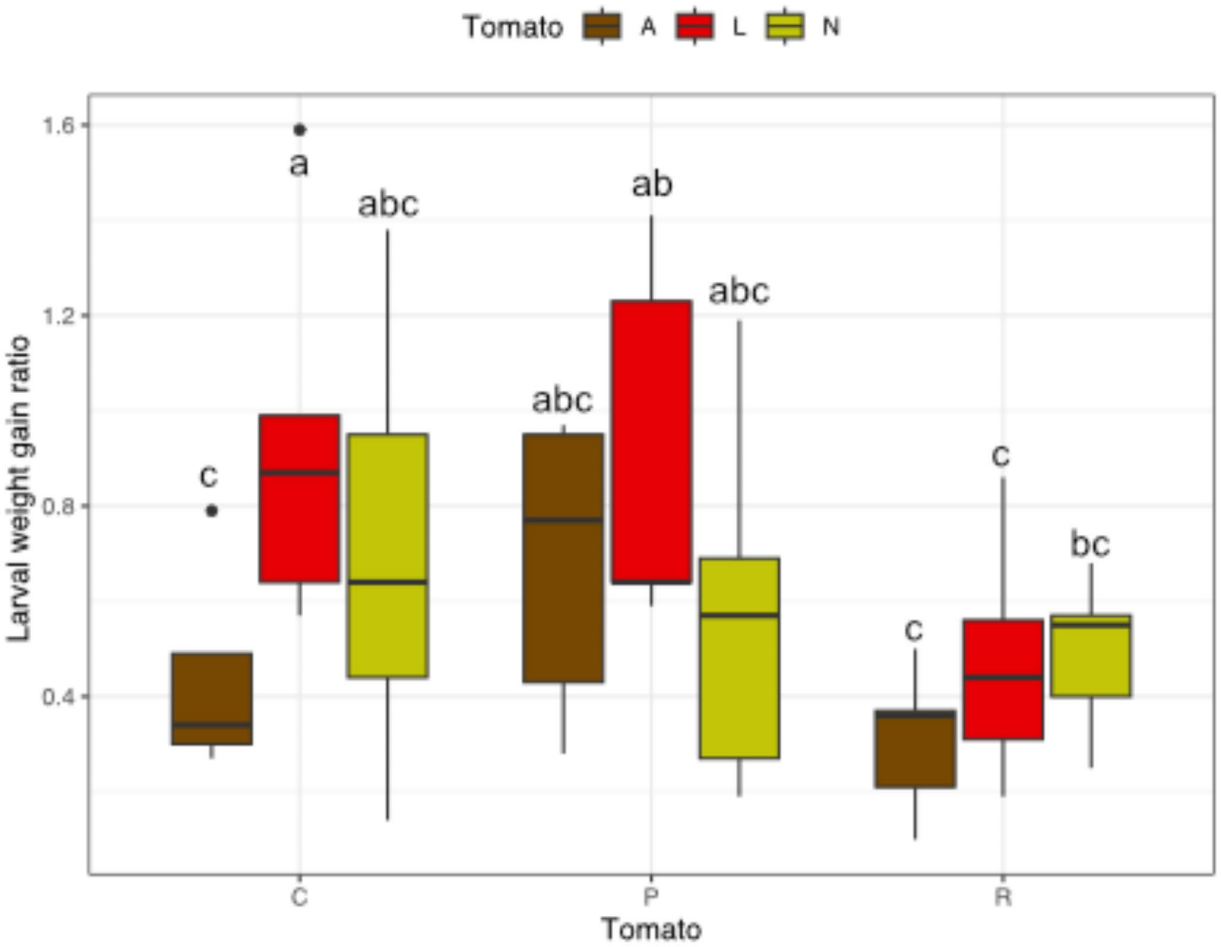
Tomato insect herbivore resistance. Barplots represent the mean of the Larval Weight Gain Ratio of the caterpillars among tomato species (A: *Solanum arcanum*, L: *Solanum lycopersicum*, N: *Solanum neorickii*) and microbial treatments (C: control; P: *Pseudomonas protegens*; R: *Rhizophagus irregularis*) for each treatment combinations. Error bars show standard deviation. Letters above boxplots indicate significant differences among tomato species and microbe treatments (Tukey HSD test, *p* < 0.05)

*Effect of tomato species and microbial treatments on herbivore resistance -* We observed that the performance of the herbivore *C. chalcites* was barely affected by the tomato species (p-value = 0.056, Table 1), nonetheless, in average for all microbial treatments, caterpillars fed on *S. lycopersicum* had a weight gain 23% higher than when fed on *S. neorickii*, and 62% higher than when fed on *S. arcanum* (Fig. 4). We also found that the microbial treatment significantly affected the performance of the herbivore (p-value = 0.03, Table 1). Specifically, for *S. lycopersicum*, the larval weight gain was 47.6% lower for AMF-inoculated than PGPR-inoculated plants, and 49.3% less than the control plants. Generally, the herbivore performance followed the same trend on *S. neorickii*, while on *S. arcanum* caterpillars grew slightly better on the PGPR-inoculated plants.

*Untargeted metabolomics across tomato plants -* Overall, we found that *S. pennellii* roots produced the highest diversity of metabolic features, with 987 unique features in average, followed by *S. arcanum* with 819, *S. neorickii* with 732, and *S. lycopersicum* with the lowest diversity of 642 features in average (Table S1, Fig. S4). For leaves, features’ diversity followed the same gradient, with *S. pennellii* producing 1259 different features in average, followed by *S. arcanum* with 1019, *S. neorickii* with 885, and *S. lycopersicum* with the lowest diversity of 633 features in average. In total, we detected 5043 unique features across all organs and tomato species.

**Fig. 5:**
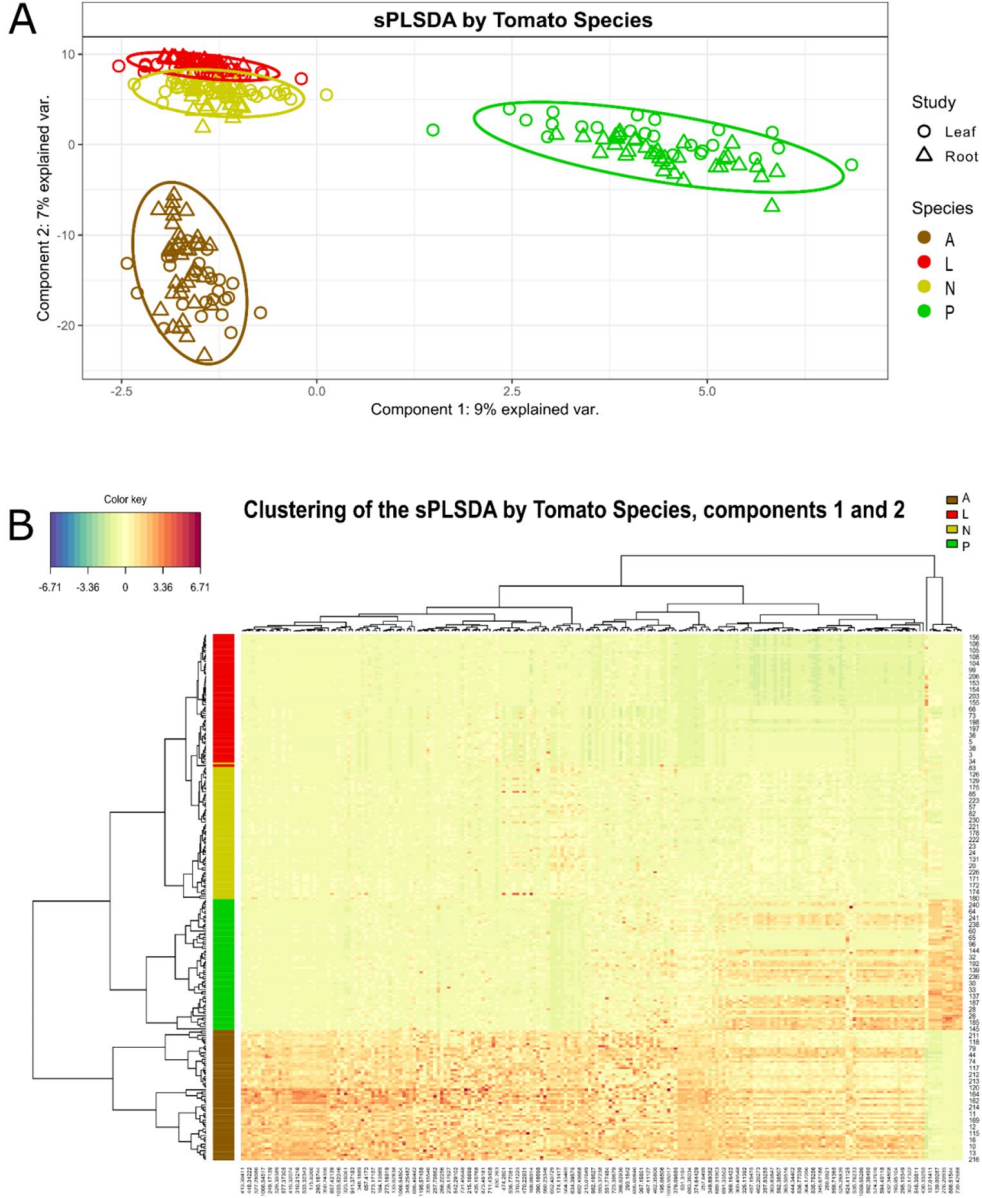
Effect of tomato species on the metabolome. **A**: Sample plot representing the projection of from both leaf and root samples onto the global variates (dimension 1 and 2) yielded by MINT sPLS-DA (2 components, 10 and 200 features respectively). Each point corresponds to an individual (leaf samples represented by rounds and roots samples represented by triangles). Ellipses represent 95% confidence intervals around tomato species (A: *Solanum arcanum*, L: *Solanum lycopersicum*, N: *Solanum neoricki*, P: *Solanum. pennellii*). **B**: Hierarchical clustering of the selected features for the tomato species s-PLSDA. Agglomerative hierarchical clustering of the 2 components for both leaf and root samples was plotted using the ‘euclidean’ distance as the similarity measure and ‘complete’ methodology as clustering method. The resulting heatmap contains the samples in rows and the features in columns, with red indicating up abundance and green down abundance. On the top of the heatmap, chemical features are clustered. On the left-hand side of the heatmap, tomato species clustering is shown using the same treatment colours as above

The PLS-DA analyses separated species based on the cumulative root and leave metabolomes (Fig. 5A), with the two first components explaining a total of 16% of the metabolome variability due to the identity of the tomato species based on 10 molecular features for the first component, and 200 features for the second component, selected through the tuned MINT s-PLSDA model (Fig. 5A, Table S2, Fig. S5 and S6). Specifically, *S. pennellii* was mostly separated from the other three species along the first axis of the PLSDA, while the three other species are separated best along the second axis, with *S. lycopersicum* ‘moneymaker’ and *S. neorickii* being the most similar in their metabolomic make-up, independently of organ (root versus shoots) type (Fig. 5A). Nonetheless, the hierarchical clustering mostly separated *S. lycopersicum* and *S. neorickii*, while including all microbial treatments (Fig. 5B). Models including less features, as well as overloaded models with more features, tested during analysis, showed less optimal clustering.

*Effects of microbial inoculation on local and systemic tomato plant metabolome -* In roots, plants of all species, besides *S. pennellii*, increased the diversity of metabolic features when inoculated with soil microbes (Table S1, Fig. S4A). Overall, plant inoculated with *P. protegens* had the highest diversity with 826 different features in average, followed by *R. irregularis* with 785, and then the control with the lowest diversity of 773 features in average. On the contrary, for the leaf metabolome, we found little effect of soil microbes, but with a general tendency of decreasing chemical diversity when soil microbes were added, particularly with AMF (Table S1, Fig. S4B).

**Fig. 6:**
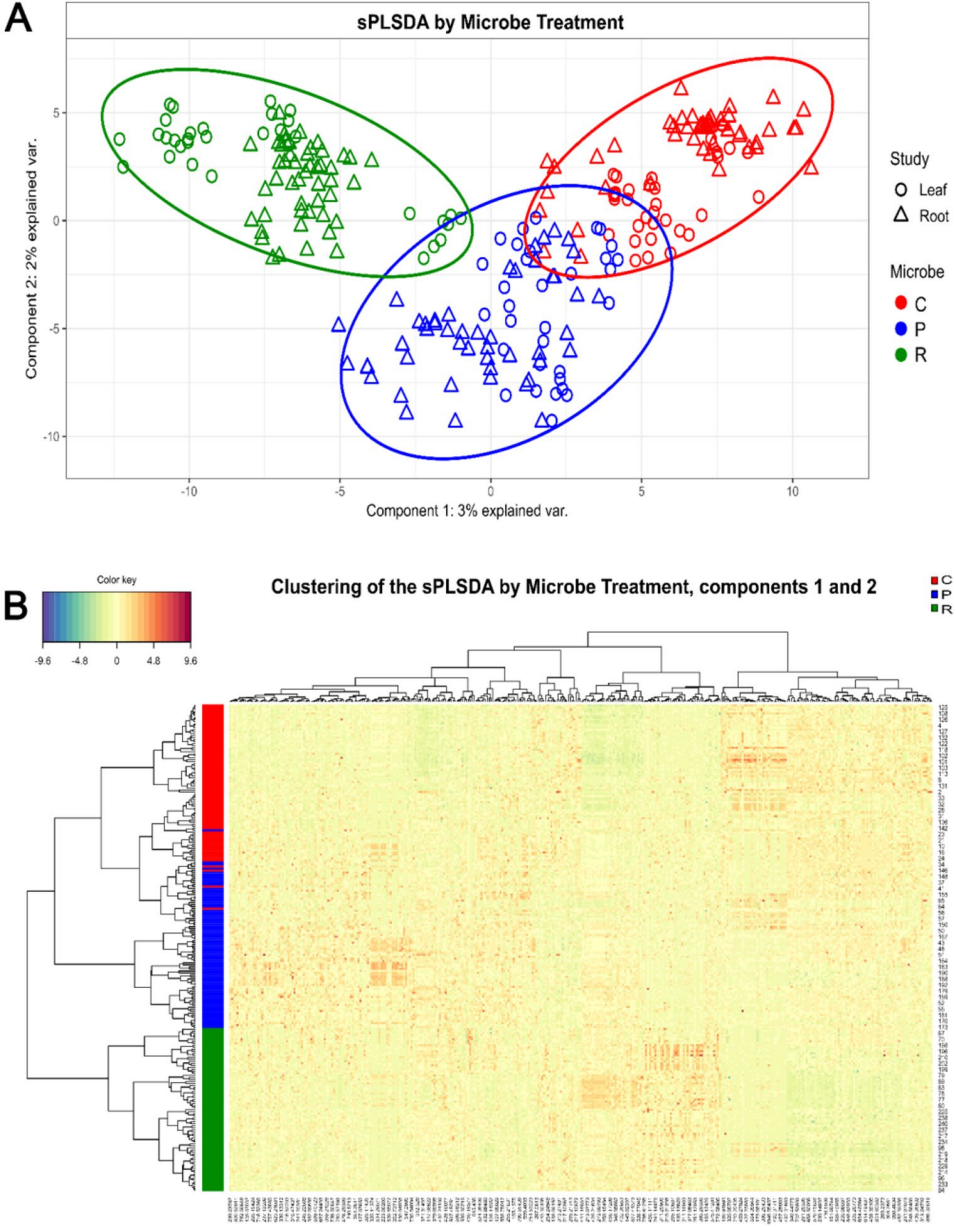
Effect of microbe treatments on the metabolome. **A**: Sample plot representing the projection of from both leaf and root samples onto the global variates (dimension 1 and 2) yielded by MINT sPLS-DA (2 components, 200 features in each). Each point corresponds to an individual (leaf samples represented by rounds and roots samples represented by triangles). Ellipses represent 95% confidence intervals around microbial treatments (C: control; P: *Pseudomonas protegens*; R: *Rhizophagus irregularis*). **B**: Hierarchical clustering of the selected features for the tomato species s-PLSDA. Agglomerative hierarchical clustering of the 2 components for both leaf and root samples was plotted using the ‘euclidean’ distance as the similarity measure and ‘complete’ methodology as clustering method. The resulting heatmap contains the samples in rows and the features in columns, with red indicating up abundance and green down abundance. On the top of the heatmap, chemical features are clustered. On the left-hand side of the heatmap, microbial treatments clustering is shown using the same treatment colours as above

The two first components of the sPLSDA explained a total of 5% of the metabolome variability due to the microbial inoculation based on 200 molecular features for the first component and 200 features for the second component, selected through the tuned MINT s-PLSDA model (Fig. 6A, Table S3, Fig. S7, and S8). Nonetheless, the analysis discriminated the samples based on the microbial treatments, with the AMF treatment separating samples from control plants more than the PGPR treatment (Fig. 6A). Accordingly, the three microbial treatments produced three distinct metabolomic profiles, as shown by the hierarchical clustering including the 400 features from components 1 and 2, for both root and leaf samples (Fig. 6B). Models including less features, as well as overloaded models with more features, tested during analysis, showed less optimal clustering. The high number of features needed by the model indicated a complex modification of metabolome.

## Discussion

We explored plant–microbe–leaf herbivore interactions in domesticated tomato and wild tomato relatives. We found that domestication generally modified plant growth and plant responses to microbial inoculation. The effect on growth and defence was dependent to the tomato species and related to the type of the microbial inoculum, either AMF or PGPR. Interestingly, PGPR-inoculated plants were taller and heavier, and they were not less susceptible to caterpillar herbivory. Contrarily, AMF-inoculated plants had comparable height and biomass than the control plants but were more resistant to herbivory, especially for the domesticated species *S. lycopersicum* var. *lycopersicum* cv. ‘MoneyMaker’.

*Impact of tomato species on microbial colonization -* We measured AMF colonization across species and found that the level of colonization decreased in the order S. *lycopersicum*,* S. arcanum*, *S. neorickii* and *S. pennellii*. Interestingly, this classification mimics the phylogenetic relationship of the *Solanum* section *Lycopersicon* clade (Aflitos et al. 2014), in which *S. lycopersicum* is the most derived species, while *S. pennellii* the most ancient species. Therefore, it seems that at speciation, tomato plants tend to acquire ever increasing ability to associate with AMFs. These observations are in opposition with recent findings on the genus *Plantago*, in which more recently derived species were less associated with AMFs than older species (Formenti et al. 2023). This would suggest that evolutionary patterns of plant-AMF association are somewhat phylogenetically constrained, but the direction of the effect is clade-dependent. Future work, involving a higher number of cultivars, and looking at the expression of genes responsible for AMF-plant interaction might help teasing this apart. Moreover, different theories have been put forward about to what extent domestication can impact the colonization of domesticated crops by AMF (Chang et al. 2022), and one common prediction is that domestication should lower the ability to associate with AMFs, since domesticated crops do not need to forage for food as much as wild species (Liu et al. 2020). Yet, the very few studies exploring this question showed important differences depending on the crop species. For instance, when comparing *S. lycopersicum* and its closely wild relative *S. pimpinellifollium*, no effect of the species was found on colonization at low phosphorus supply, but at high phosphorus supply, the mycorrhization was slightly higher in the wild species (Martín-Robles et al. 2018). Our study, performed under normal phosphorus fertilization, complete these findings with an even more marked effect of the tomato species, likely because our work covers species with a stronger phylogenetic distance (Aflitos et al. 2014).

For the bacterial inoculation of *P. protegens*, previous studies on *S. lycopersicum* (Lax et al. 2013; Aiello et al. 2017; Zhao et al. 2021) and other crops (Flury et al. 2016; Chiriboga. et al. 2018) showed a good and predictable level of colonization by this PGPR when applying the liquid inoculum directly in the root zone, as done in our study. In our case, the significant effects obtained on the plant growth and defences after the targeted inoculation of this specific microbe confirmed a good colonization of the tomato plants (Figs. 1, 2 and 3). For all species, plants inoculated with *P. protegens* had belowground and aboveground biomasses increased, confirming the growth-promoting effect. To our knowledge, this effect had previously only been documented in domesticated tomato species (Gamalero et al. 2002). However, to fully validate this finding, further testing of this PGPR’s activity is necessary, as this aspect was not included in our protocol.

*Impact of domestication and microbial inoculation on plant growth -* In our study, the domesticated species distinguished itself form the three wild species regarding the growth aspect, with higher stem width, higher belowground and aboveground biomass as well as higher fruit yield (Fig. 1). This is perhaps not surprising, as domesticated species have been selected for yield optimization, including more rapid and shorter reproductive cycles than wild species (Jiménez-Gómez et al. 2007), while, on the contrary, growth and reproduction of wild species is the result of adaptive responses to their native environment, generally within the ecological context of limiting resource use and development (Patterson et al. 1978; Nakazato et al. 2008). The process of evolution across eco-climatic disruptions in South America is the principal theory explaining phenotypic and genetic variations among wild species (Rick and Lamm 1955; Rick and Holle 1990). Recently, studies found the genetic origins of traits such as leaf development or mating system, and proved the effect of natural selection as driver of differentiation across wild tomato species (Muir et al. 2014; Landis et al. 2021). These evidences corroborate the differences of growth and fruit yield that we observed in our study with the agronomically selected cultivar (Roohanitaziani et al. 2020).

*Impact of domestication on alkaloids production -* The alkaloids that we measured and that had the highest concentrations in the leaves, have been described as end-products of the GAME (Glykoalkaloid Metabololism Enzymes) pathway as described by Itkin et al. (2013) and were produced in a characteristic blend for each tomato species in our study. According to Razifard et al. (2020), different chemical phenotypes, commonly believed to be the end-result of domestication, like alpha-tomatine content, appear not to be a result of any domestication process, but rather are the result of local adaptation, as levels of alpha-tomatine are highest in a single *Solanum pennellii* group (SP South Ecuadorian) and show increased variance in Peruvian *Solanum lycopersicum* var. *cerasiforme* (SLC) populations and in *Solanum lycopersicum* var. *lycopersicum* (SLL). Also, according to our predictions, we found that the domesticated species produced the lowest diversity of glycoalkaloids, based on what was detectable in the leaves. This would suggest that through selection and breeding, modern tomato cultivars may reduce their investment in defence by producing a reduced diversity of compounds, while still maintaining sufficient levels of alpha-tomatine, as a necessary chemical defence trait, already mentioned for the fruit (Rick et al. 1994; Iijima et al. 2013).

Moreover, we found wide differences between roots and leaves of the same species. For example, while *S. pennelli* produced the highest alpha-tomatine concentration in roots, the same species produced the lowest concentration in the leaves compared to the other three species tested (Figs. 2 and 3). Investigations in this regard are rare on this species, but we could advance some hypotheses. For instance, *S. pennellii*, the most ancestral species of our study (Aflitos et al. 2014; Dodsworth et al. 2016), is also known to sustain important amounts of acylsugars in leaves, which act as sticky physical defence against insect pests (Berlinger et al. 1997; Maluf et al. 2010b). Therefore, it might be that *S. pennellii* is trading off chemical and physical defences in leaves (Moghe et al. 2023), while in roots, this might not be possible. Why in general tomato species produce a different composition of alkaloids in leaves and roots remains an open question (Kim et al. 2014), but this finding has been deeply investigated for glucosinolates compounds in *Brassicaceae* (van Dam et al. 2009; Tsunoda and van Dam 2017; Touw and van Dam 2023). Also, this question has been studied for alkaloids in other *Solanaceae* related species, for instance, in *Solanum carolinense* the concentration of solasodine is much higher in the roots (Walls et al. 2005), or, in *Datura innoxia*, scopolamine is produced in roots and accumulated in shoots (Schlesinger et al. 2021), suggesting that root and shoot chemistry follows different coupling and selection regimes (Rasmann and Agrawal 2009).

*Impact of microbial inoculation on alkaloids -* For chemical plant defences, we found that *S. lycopersicum* is not impacted by any microbial inoculum for the concentration of main glycoalkaloids in the root while wild species showed small differences (Fig. 2). In the leaves, the domesticated tomato plants inoculated by either the AMF or the PGPR had higher concentrations of alpha-tomatine. The same was observed for *S. neorickii* inoculated by AMF (Fig. 3). For other compounds, when present in the plant, both microbial inocula tended to decrease concentrations and more significantly for the AMF showing that the microbial interaction can reshape the chemical defence blend of the plants. For instance, in *S. arcanum*, inoculated plants showed lower concentrations of hydroxytomatine (Fig. 3C), which may reveal the cost of the symbiosis, as we know this molecule is created at a further step than alpha-tomatine in the GAME pathway (Szymański et al. 2020; Akiyama et al. 2022). Additionally, AMF colonization rate might not be a good predictor for the strength of their effects on the plant metabolome. Indeed, despite the negligible colonization of *S. pennellii*, AMF had a strong impact on the root concentrations of glycoalkaloids (Fig. 2). AMF presence altered the metabolic profiles, but higher colonization rates were not necessarily associated with stronger effects. Therefore, AMF is known to influence the trade-off between investment in growth or chemical defence in tomato plant under stressful environment, for instance under drought, decreasing either growth or defence (Orine et al. 2022), while the PGPR is known to increase nitrogen uptake, enhancing plant growth (Pérez-Rodriguez et al. 2020; García-Villaraco et al. 2021). Overall, we conclude that domestication has generally modified the way tomato plants are interacting with microbes, for either growth and defence, and dependent to both the plant host and the beneficial microbe genotypes (Carrillo et al. 2019; Jaiswal et al. 2020).

*Impact of domestication and microbial inoculation on herbivore resistance -* Concerning plant resistance to insect herbivore attack, in accordance with the chemical defence hypothesis, that domestication result in reduced chemical defences, we predicted that larvae of *C. chalcites* would perform less well on wild tomato species compared to the cultivated one. Accordingly, for non-inoculated plants, we found that *C. chalcites* feeding on *S. arcanum* were smaller than on the other species (Fig. 4). Within the three tomato species tested in the bioassay, *S. arcanum* differentiated itself from the other species by producing higher levels of hydroxytomatine, perhaps indicating that this insect is more sensitive to the toxicity of this compound. Future targeted bioassays with artificial diets could help teasing this apart (Rasmann and Agrawal 2009). Similarly, for another major pest of tomato, *Tuta absoluta*, it was found that it grew better on the cultivated variety than on wild species as *S. peruvianum* or *S. habrochaites* (Terzidis et al. 2014). On *S. neorickii*, the published data indicates no particular resistance against the pest *T. absoluta* (Kayahan et al. 2018; Aslan and Birgücü 2022), confirming our results with *C. chalcites* for this wild species. Most herbivory tests reported in the literature were conducted with *S. pimpinnelifolium* as a wild relative in comparison to modern tomato (Turcotte et al. 2014). For instance, *Helicoverpa zea* caterpillars performed worse on *S. pimpinnelifolium* than on *S. lycopersicum*, but levels of glykoalkaloids in leaves were not quantified (Paudel et al. 2019). Moreover, as our work suggests, it seems that no single specific compound is the only responsible for insect resistance in tomato plants. Indeed, more than one hundred of putative steroidal glycoalkaloids have been screened among wild tomato species (Iijima et al. 2013; Schwahn et al. 2014). Hence, the chemical defences of tomato species seem to be dependent on complex blends of glycoalkaloids and other molecules (e.g., acyl sugars (Maluf et al. 2010a), each having different impacts on different herbivore pests (Nakayasu et al. 2020).

Concerning the impact of soil microbes on insect resistance we found that against the pest herbivore, the domesticated tomato, which showed the highest colonization by AMF, was the only species to benefit in terms of a significant reduction in the caterpillar performance. Thus, the better activation of MIR on *S. lycopersicum* annihilated the difference in caterpillar performance between the modern cultivar and the two wild species. As defence stimulator in our study, *P. protegens* had no effect to trigger plant defence against caterpillars of *C. chalcites*, while it is known to have direct entomopathogenic effects on insects like *Manduca sexta* or *Galleria mellonella* (Péchy-Tarr et al. 2008) and is used as biocontrol agent against fungal pathogens (Chandrasekaran et al. 2016; Roca-Couso et al. 2021).

*Impact of microbial inoculation on tomato plants’ metabolomes -* Untargeted metabolomics studies on domestication remain rather rare, particularly in tomato, for which most analyses concern the fruit taste and smell (Zhu et al. 2018; Razifard et al. 2020). Using constrained multivariate analyses, we were able to discriminate tomato species based on their leaf and root metabolomics signatures. Moreover, the tuned PLS-DA model could also discriminate the three different microbial treatments despite the mixed strong effect of the tomato species in the dataset. This latter result is rather unexpected, as we would have predicted the species-level variation to be strong enough to annihilate potentially rather smaller effects of the microbes on the metabolomes. On the other hand, we show that a microbe-based chemical signature is detectable at the whole metabolome scale, and that a complex model based on the abundance of several hundreds of features can cluster tomato plants from highly different genetic origins thanks to their rhizosphere microbiome. Unfortunately, the Data Independent Acquisition (DIA) nature of the mass spectrometry data did not allows to perform meaningful identification of molecules from the obtained chemical features (Tsugawa et al. 2015). Therefore, future work with other LC-MS modes (e.g., DDA) is required to pinpoint to unique metabolomic features that best discriminated species and microbial treatments. Nonetheless, our results attest that basal plant growth and systemic resistance to foliar pests are interactively affected by domestication and microbial inoculation. This conclusion might also help to explain why the efficacy of beneficial microbes is often so variable in field trials with different landraces or trade cultivars (Jaiswal et al. 2020).

*Conclusion****-*** This study, combining plant-growth traits as well as targeted and untargeted metabolomics, aimed to bring new knowledge on the effects of AMF and PGPR inoculation on domesticated and wild tomato species in terms of plant growth and insect resistance. Generally, we showed that the PGPR tended to increase productivity, while AMF tended to decrease it, and that AMF increase insect resistance, particularly for the commercial variety. Additionally, we found that each microbial inoculum influenced the metabolome of both modern cultivar and wild relative species, in a species-dependent manner. *S. lycopersicum* was more likely to be colonized by AMF than its wild relatives. Our results provide researchers with tomato chemical traits that can be investigated to understand the variable effects of commercial microbial inoculum on different tomato species and modern crop cultivars. Further effort is required to decipher the mechanisms underlying these changes and their implications for the biocontrol and productivity of tomato crop.

## Electronic Supplementary Material

Below is the link to the electronic supplementary material.

Supplementary Material 1

## Data Availability

No datasets were generated or analysed during the current study.
