# Supplementary material for: Microbial Interactions Influence the Chemical Defense of Wild and Cultivated Tomato Species
Source: J Chem Ecol. 2025 Apr 7;51(2):47. doi: 10.1007/s10886-025-01598-y (PMC11976755; doi:10.1007/s10886-025-01598-y)
Supplement: Supplementary file 1 — Supplementary Material 1 [file 10886_2025_1598_MOESM1_ESM.docx]

**Supplementary material for**

**Arbuscular mycorrhizal fungi and bacteria shape the chemical defence of modern and wild tomato species**

Dimitri Orine, Haymanti Saha, Gaetan Glauser, Arjen Biere, Sergio Rasmann

**Methods S1**

*Plant secondary metabolites analyses* - Leaf samples of eight plant replicates and root samples of twelve plant replicates were extracted following a usual procedure. Briefly, freeze-dried leaf material was ground to powder, and 20 mg of each sample was extracted with 2 mL of an extraction solution consisting of HPLC grade MeOH:milliQ H_2_O (80:20, v/v %) acidified with 0.1% glacial acetic acid. Samples tubes holding two glass beads were shaken in a Retsch MM400 grinder at 30 Hz for 5 min, followed by 5 min centrifugation at 12000 rpm. Clear supernatants were used for the HPLC-MS/MS processing.

The analysis of the chemical extracts was performed by ultra-high-performance liquid chromatography - quadrupole time-of-flight mass spectrometry (UHPLC-QTOFMS) using an Acquity UPLC™ system coupled to a Synapt G2 QTOF mass spectrometer (Waters, Milford, MA, USA) controlled by Masslynx 4.1. The separation was performed on a Waters Acquity HSS T3 column (100 × 2.1 mm i.d., 1.8 μm particle size) thermostated at 25 °C. The separation was performed in gradient mode using water containing 0.05% formic acid as mobile phase A and acetonitrile containing 0.05% formic acid as mobile phase B. The gradient started at 0% B and increased to 50% B in 7.5 min, then to 100% B in 2.5 min, followed by a hold at 100% B for 2 min and re-equilibration at 0% B for 4 min. The injection volume was 1 μl. MS detection was performed using data-independent acquisition (DIA). The Synapt G2 was operated in positive electrospray using alternate scans at low (4 eV) and high (8-50 eV ramp) collision energies. An *m/z* range of 50-1200 Da was covered for a total cycle time of 0.45 s. Source parameters were: temperature 120°C, capillary voltage 2.5 kV, cone voltage 25V, desolvation gas flow and temperature 900 L/h and 400°C, respectively, cone gas flow 50 L/h. A 500 ng/ml solution of the synthetic peptide leucine-enkephaline in water:acetonitrile:formic acid (50:50:0.1) was infused constantly into the mass spectrometer as internal reference to ensure accurate mass measurements (<2ppm).

**Methods S2**

*Glycoalkaloids quantification* - For targeted analyses, the glycoalkaloids alpha-tomatine, hydroxytomatine, dehydroxytomatine, acetoxytomatine (I), acetoxytomatine (II), and dehydroacetoxytomatine were identified based on their molecular formula and fragmentation spectra at high energy. The TargetLynx XS module of the MassLynx software (Waters, Milford, MA, USA) was used to quantify the amount of glycoalkaloids based on a calibration curve made with a chemical standard of pure alpha-tomatine from Sigma-Aldrich (St Louis, USA). For quantification, peaks areas were automatically integrated by the software with a 0.1 min chromatographic window centred on the retention time of each component and a 0.02 Da mass window centred on the (M + H^+^) ion. The glycoalkaloids concentration was expressed in μg per gram of dried plant as equivalent of alpha-tomatine.

**Methods S3**

*Untargeted metabolomics data processing* - For the processing of the data in untargeted mode, the centroided and lockmass corrected raw data were converted to mzXML using MSConvert (ProteoWizard). The Mass Spectrometry-Data Independent AnaLysis (MS-DIAL) software was used to perform assignment of m/z groups to features (Tsugawa *et al.*, 2015; Tada *et al.*, 2020). The processing pipeline parameters were as follows; mass accuracy for data collection was settled at 0.01 Da for MS1 and 0.025 Da for MS2; peak detection was performed with a smoothing level of 3 scans and a minimum peak width of 5 scans; MS2 deconvolution used a 0.5 sigma window value and an amplitude ratio of 40 for MS/MS abundance cut off; alignment parameters were set at 0.17 min for retention time tolerance and 0.015 Da for MS1 tolerance. Metabolomics data from root and leaf samples were analysed in a common batch to allow comparisons and further joint analyses. The matrix of features abundances was then used as a presence/absence matrix for the feature’s diversity analysis. An additional step of missing-value interpolation was performed with MS-DIAL as described by Tsugawa *et al.*(2015) to fit with PLSDA requirements. The Data Independent Acquisition (DIA) nature of the mass spectrometry data compromise efficient identification of molecules from the chemical features of non-lipidic compounds but are greatly appropriated for estimating compound diversity and multivariate statistics (Tsugawa *et al.*, 2015)**.**

**Methods S4**

*Multivariate analyses* - The MINT sPLS-DA method that generalizes sPLS-DA while accounting for study-specific effects (root or leaf samples) (Rohart *et al.*, 2017a) was performed following standard procedures (Rohart *et al.*, 2017a,b). MINT simultaneously integrates the samples from different tissues (root and leaf) consisting of complementary metabolomic datasets and selects the most discriminant variables to classify. Next, MINT seeks for a common projection space for all studies that is defined on a small subset of discriminative variables and that display an analogous discrimination of the samples across studies. The identified variables share common information across all studies and therefore represent a reproducible signature that helps characterising biological systems. Ten-fold cross-validation was used to select the number of PLS-DA components, and the number of variables for best clustering. Loadings weights explain the direct regression coefficients used to define the latent components and show the major discriminants between the groups analysed. The final MINT model was then fitted to the data, and the classification performance was estimated using the *perf* function and 10-fold cross-validation repeated 10 times. Graphical display of the discriminative features signature identified by MINT were output as heatmap using *clustered image map* function.

**Table S1.** Two-way interaction ANOVA table for measuring the effect of drought (two levels) and AMF treatments (four levels) on chemical features diversity.

| **Response variable** | **Treatment** | **Df** | **SumSq** | **MeanSq** | **F value** | **p-value** |
| --- | --- | --- | --- | --- | --- | --- |
| Root features number (log) | Microbe (M) | 2 | 0.142 | 0.0708 | 31.06 | <0.001 |
|  | Tomato species (S) | 3 | 3.572 | 1.1906 | 522.57 | <0.001 |
|  | M x S | 6 | 0.165 | 0.0276 | 12.1 | <0.001 |
|  | Residuals | 132 | 0.301 | 0.0023 |  |  |
|  |  |  |  |  |  |  |
| Leaf features number (log) | Microbe (M) | 2 | 0.075 | 0.0376 | 17.278 | <0.001 |
|  | Tomato species (S) | 3 | 5.96 | 1.9868 | 912.571 | <0.001 |
|  | M x S | 6 | 0.023 | 0.0039 | 1.778 | 0.113 |
|  | Residuals | 86 | 0.187 | 0.0022 |  |  |

**Table S2.** **Statistics of the MINT sPLSDA by tomato species.** Area Under the Curve (AUC) and associated p-value, for component 1 and both components 1 and 2, for the root contribution, the leaf contribution and the global model as shown in Figure 5, S5, S6.

| Component 1 | Root | | Leaf | | Global | |
| --- | --- | --- | --- | --- | --- | --- |
|  | AUC | p-value | AUC | p-value | **AUC** | **p-value** |
| A vs Other(s) | 0.7006 | 3.20E-04 | 0.6396 | 4.05E-02 | **0.5908** | **0.039** |
| L vs Other(s) | 0.8807 | 8.60E-12 | 0.6334 | 5.02E-02 | **0.6539** | **0.003** |
| N vs Other(s) | 0.4187 | 1.45E-01 | 0.7153 | 1.18E-03 | **0.5175** | **0.682** |
| P vs Other(s) | 1 | 0.00E+00 | 1 | 2.20E-13 | **0.7626** | **<0.001** |
|  |  |  |  |  |  |  |
| Component 1 and 2 | Root | | Leaf | | **Global** | |
|  | AUC | p-value | AUC | p-value | **AUC** | **p-value** |
| A vs Other(s) | 1 | 0 | 1 | 2.20E-13 | **0.756** | **<0.001** |
| L vs Other(s) | 1 | 0 | 0.8767 | 3.26E-08 | **0.736** | **<0.001** |
| N vs Other(s) | 0.6667 | 0.002793 | 0.7837 | 1.93E-05 | **0.6153** | **0.007** |
| P vs Other(s) | 1 | 0 | 1 | 2.20E-13 | **0.7626** | **<0.001** |

**Table S3.** **Statistics of the MINT sPLSDA by microbial inoculum,** Area Under the Curve (AUC) and associated p-value, for component 1 and both components 1 and 2, for the root contribution, the leaf contribution and the global model as shown in Figure 6, S7, S8.

| Component 1 | Root | | Leaf | | Global | |
| --- | --- | --- | --- | --- | --- | --- |
|  | AUC | p-value | AUC | p-value | **AUC** | **p-value** |
| C vs Other(s) | 0.9889 | 0 | 0.8653 | 3.87E-09 | **0.7347** | **<0.001** |
| P vs Other(s) | 0.5054 | 0.9156 | 0.5852 | 1.73E-01 | **0.5121** | **0.759** |
| R vs Other(s) | 0.9944 | 0 | 0.9492 | 4.39E-13 | **0.7467** | **<0.001** |
|  |  |  |  |  |  |  |
| Component 1 and 2 | Root | | Leaf | | **Global** | |
|  | AUC | p-value | AUC | p-value | **AUC** | **p-value** |
| C vs Other(s) | 0.995 | 0 | 0.8625 | 5.08E-09 | **0.7327** | **<0.001** |
| P vs Other(s) | 0.997 | 0 | 0.9242 | 1.14E-11 | **0.7421** | **<0.001** |
| R vs Other(s) | 1 | 0 | 0.972 | 2.71E-14 | **0.7525** | **<0.001** |

**Figure S1.** **Geographical distribution of the three wild tomato species tested in this study**. Top panel (A) shows the a principal component analysis (PCA) of all the 19 bioclimatic variables (retrieved from <https://chelsa-climate.org/bioclim/>) for describing the climate of occurrence observations of each species. Bottom panels (B, C, D) show the maps with occurrence data retrieved from gbif.org.


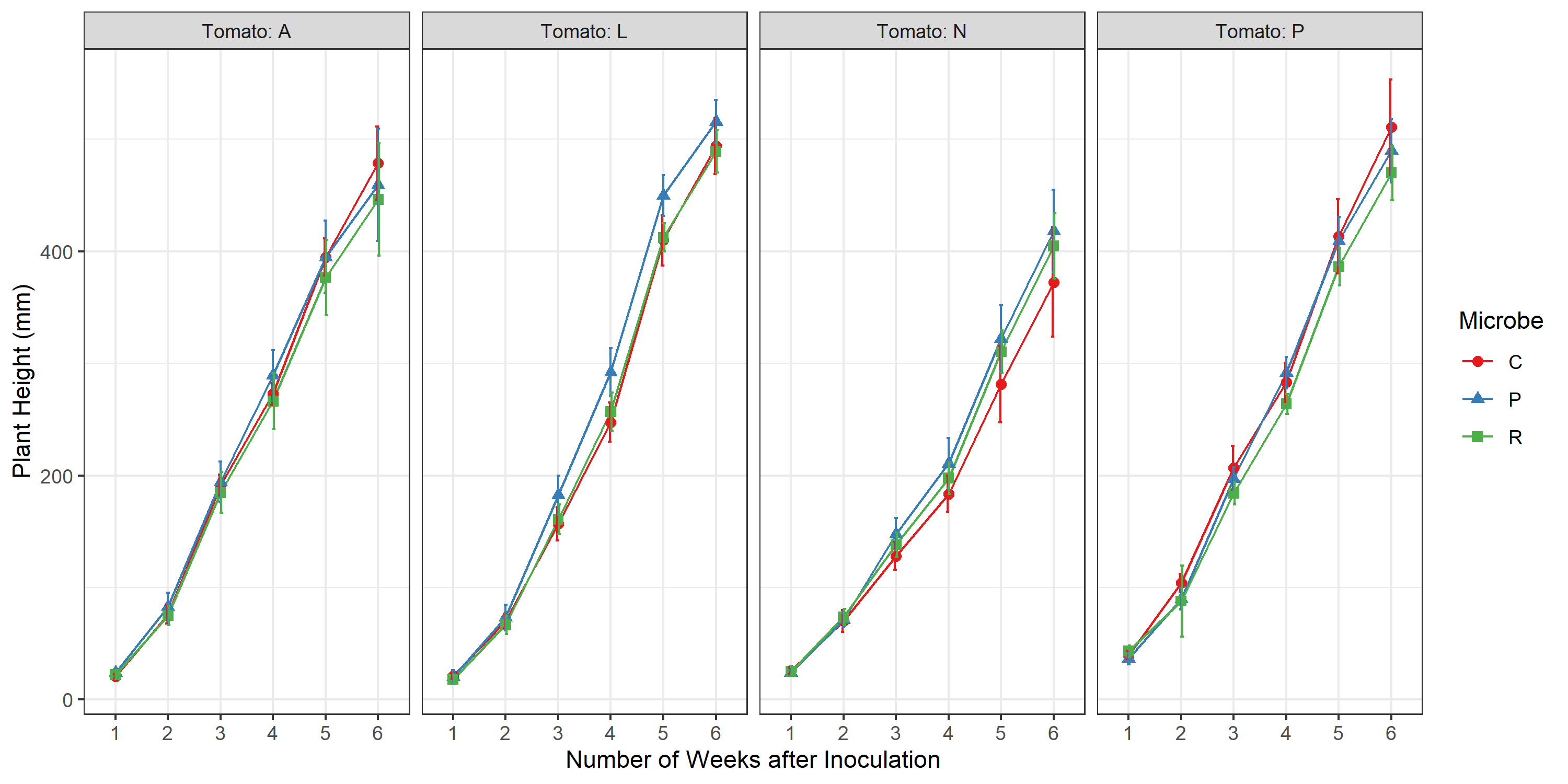


**Figure S2. Growth of four tomato species.** Shown are growth curves based on plant height from 1 week to 6 weeks after inoculation of the microbial treatments (C: control; P: *Pseudomonas protegens*; R: *Rhizophagus irregularis*) for the four tomato species (A: *Solanum arcanum*; L: *Solanum lycopersicum*; N: *Solanum neorickii*; P: *Solanum pennellii*). The error bars represent the standard deviation among the 12 replicates for each time point.


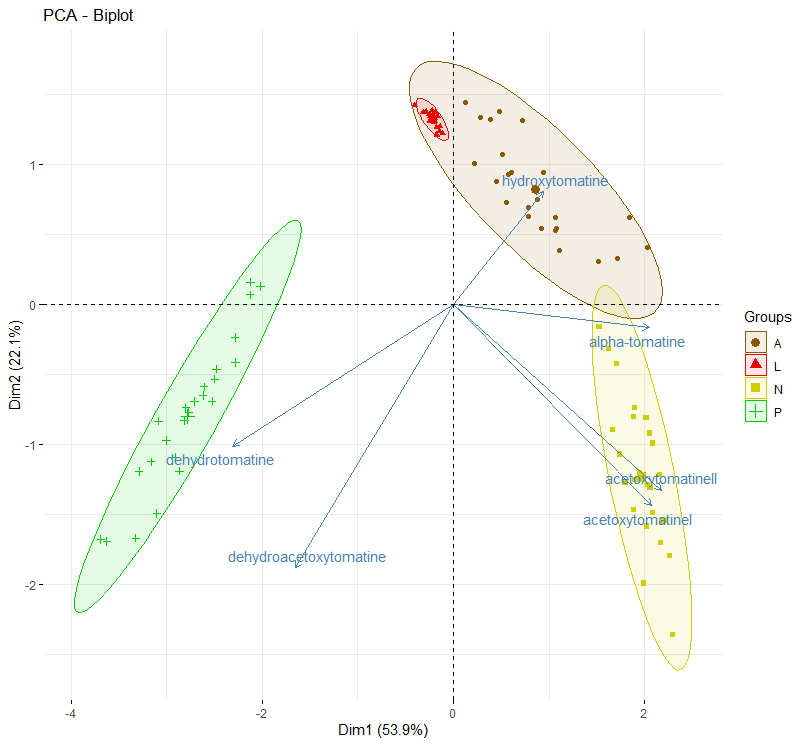


**Figure S3. Tomato chemical defences** Principal Component Analysis (PCA) ordination plots for the concentration of targeted glykoalkaloids in leaves (alpha-tomatine, dehydrotomatine, hydroxytomatine, acetoxytomatine (I), acetoxytomatine (II), dehydroacetoxytomatine). Ellipses represent 95% confidence intervals around three tomato species (A: *Solanum arcanum*, L: *Solanum lycopersicum*, N: *Solanum neorickii, P: Solanum pennellii*).


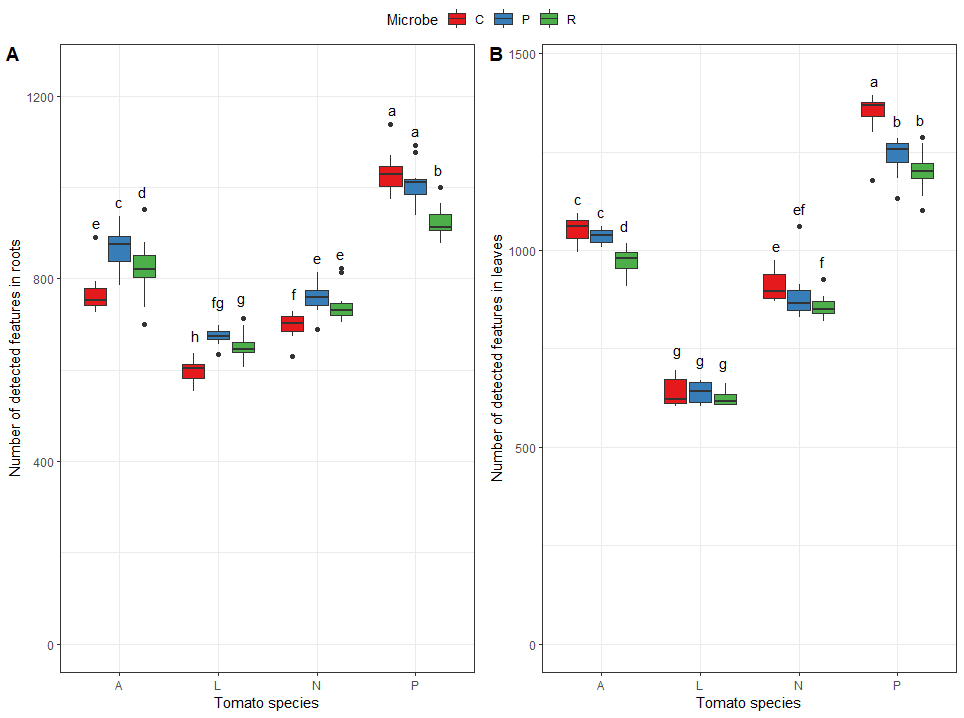


**Figure S4. Tomato species and microbe effects on chemical features diversity.** Boxplots show the number of detected features in roots (A on the left) and leaves (B on the right) for tomato species (A: *S. arcanum*; L: S. *lycopersicum*; N: *S. neorickii*; P: *S. pennellii*) and microbial treatments (C: control; P: *Pseudomonas protegens*; R: *Rhizophagus irregularis*). Letters above boxplots indicate significant differences among tomato species and microbe treatments (Tukey HSD test, *p* < 0.05). Boxplots represent, from bottom to top, minimum, first quartile, median, third quartile and maximum, and dots represent the outliners.


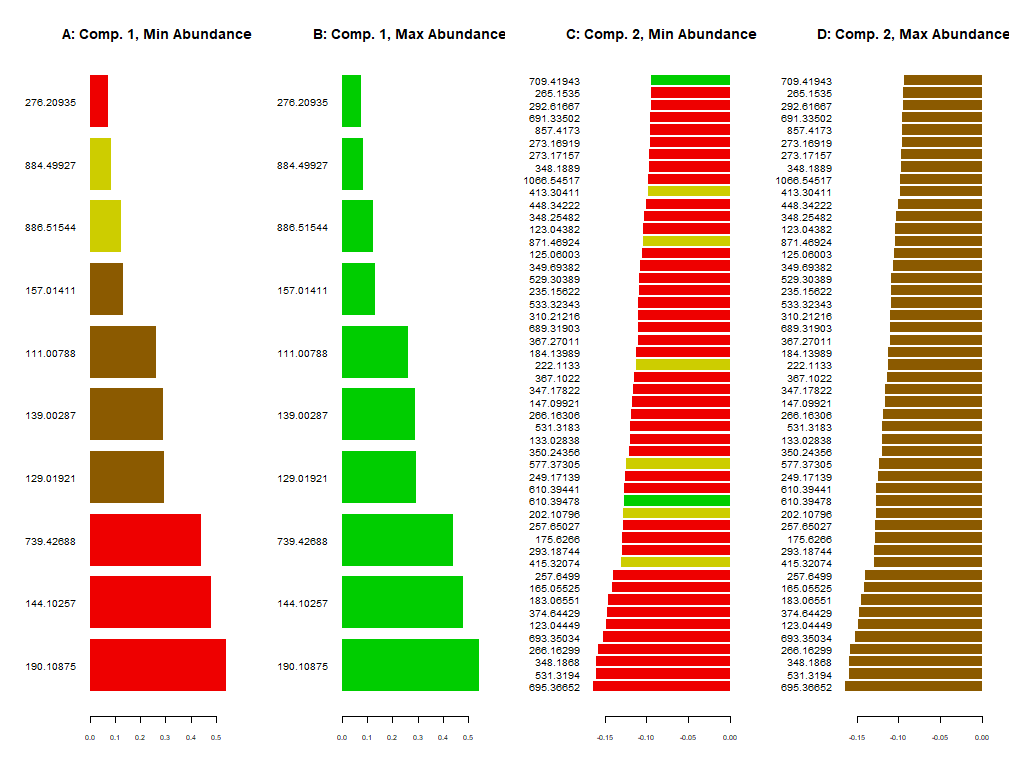


**Figure S5. Loading weights of the MINT sPLSDA by tomato species.** The bars represent the weight of the loading vectors, depicting direct coefficients maximizing the covariance between features and species, used by the model for selecting the most discriminant features affecting the clustering in each component. The colours represent the tomato species: red: *S. lycopersicum*, brown: *S. arcanum*, yellow: *S. neorickii*, green: *S. pennellii*. Numbers on the left are the m/z values (in Dalton) of the fragment identified as parental ion in the MS2 feature spectrum. Scales at the bottom indicate the weight value of loading vectors. For each of the 10 features of Component 1: **A** shows the tomato species with the minimum abundance and **B** shows the tomato species with the maximum abundance. For each of the 50 most important features of Component 2: **C** shows the tomato species with the minimum abundance and **D** shows the tomato species with the maximum abundance.


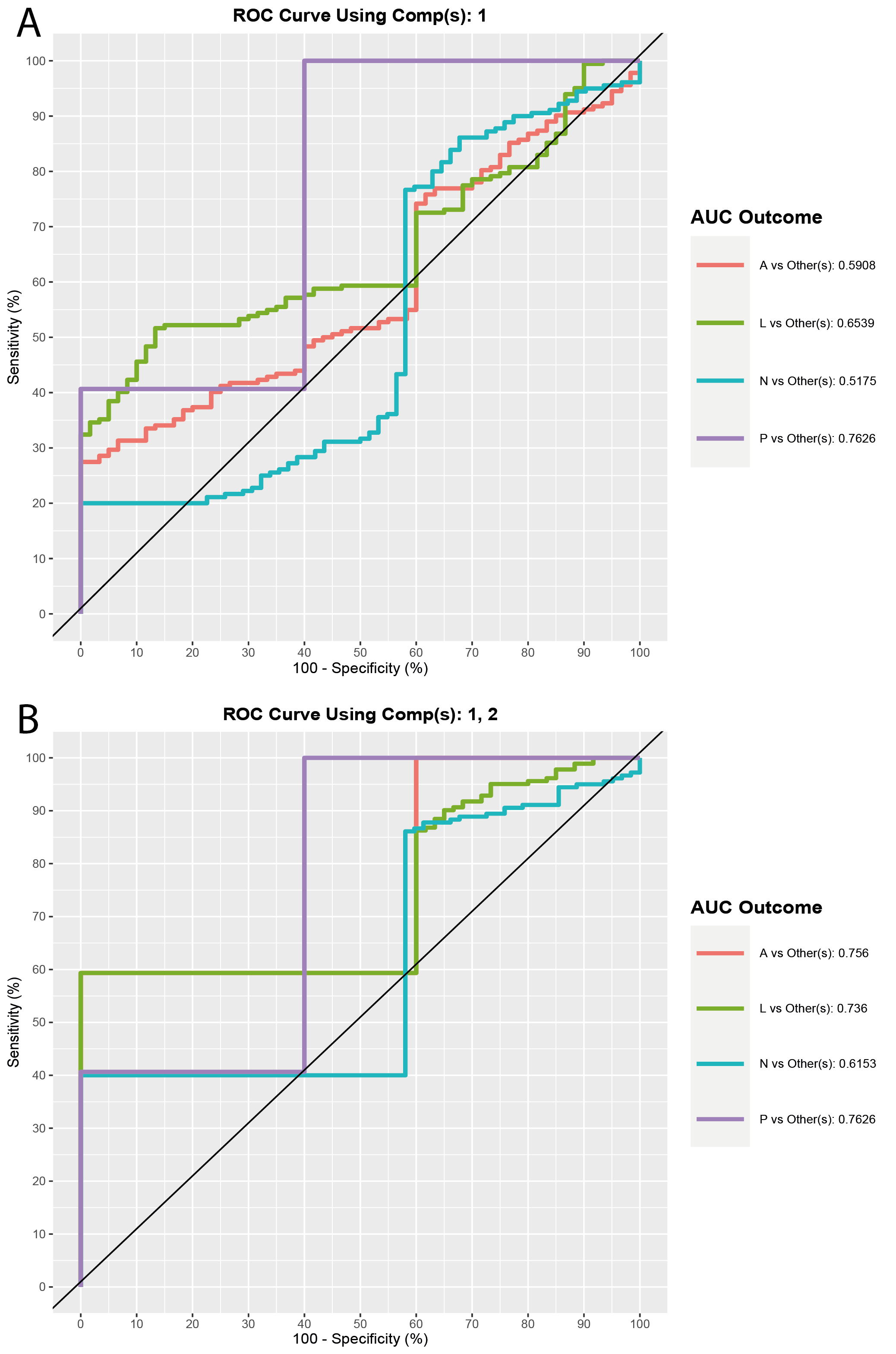


**Figure S6. ROC curve and AUC from the MINT sPLSDA by tomato species, Components 1 and 2.** The graphs show a visualisation of classification performance when undergoing the LOGOCV (Leave One Group Out Cross Validation) procedure on the model. The Receiver Operator Curve (ROC) depicts how the Sensitivity (True Positive Rate) and the 100-Specificity (False Positive Rate) change as the classification threshold changes in the MINT sPLSDA by microbial inoculum performed on the chemical features abundance data. Numerical output is the Area Under the Curve (AUC) for each ‘one vs. other’ class comparison that are performed for this component. **A:** The curves show, for component 1, ROC averaged across one-vs-all comparisons.  **B:** The curves show, for component 1 and 2, ROC averaged across one-vs-all comparisons. As PLSDA models are built iteratively (component 2 depends on built component 1), thus it makes sense to plot both components and to look how the error rate decreases when the number of components increases.


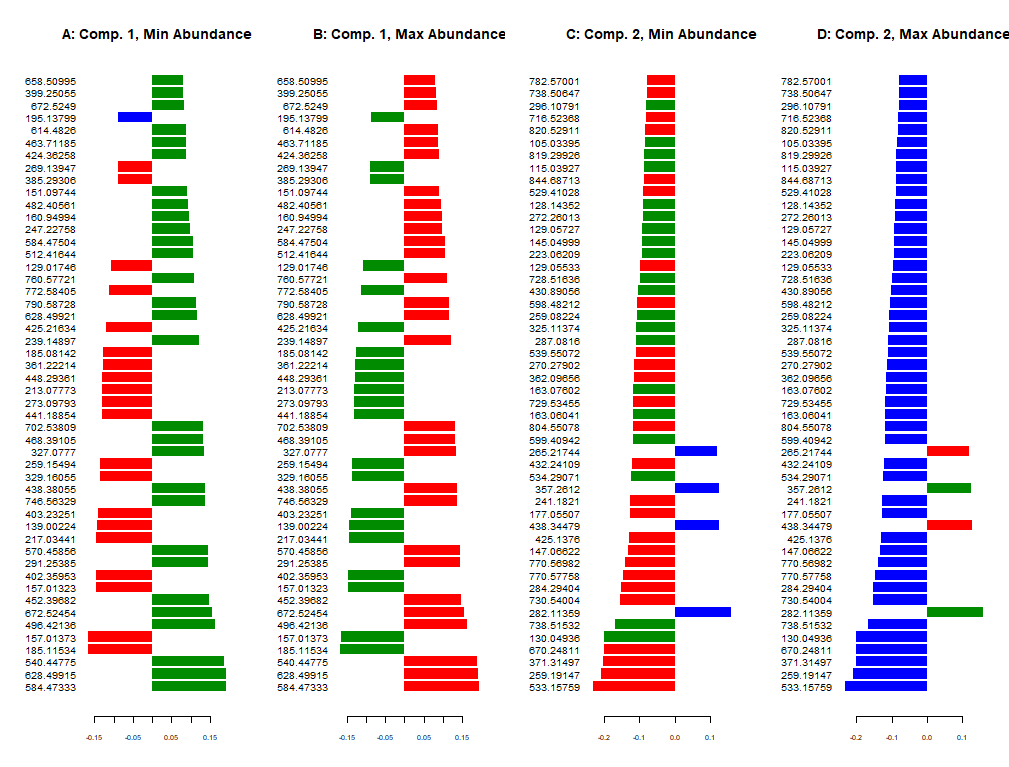


**Figure S7. Loading weights of the MINT sPLSDA** **by microbial inoculum.** The bars represent the weight of the loading vectors, depicting direct coefficients maximizing the covariance between features and species, used by the model for selecting the most discriminant features affecting the clustering in each component. The colours represent the microbial inoculum: red: Control, blue: *P. protegens*, green*: R. irregularis*. Numbers on the left are the m/z values (in Dalton) of the fragment identified as parental ion in the MS2 feature spectrum. Scales at the bottom indicate the weight value of loading vectors. For each of the 50 most important features of Component 1: **A** shows the tomato species with the minimum abundance and **B** shows the tomato species with the maximum abundance. For each of the 50 most important features of Component 2: **C** shows the tomato species with the minimum abundance and **D** shows the tomato species with the maximum abundance.


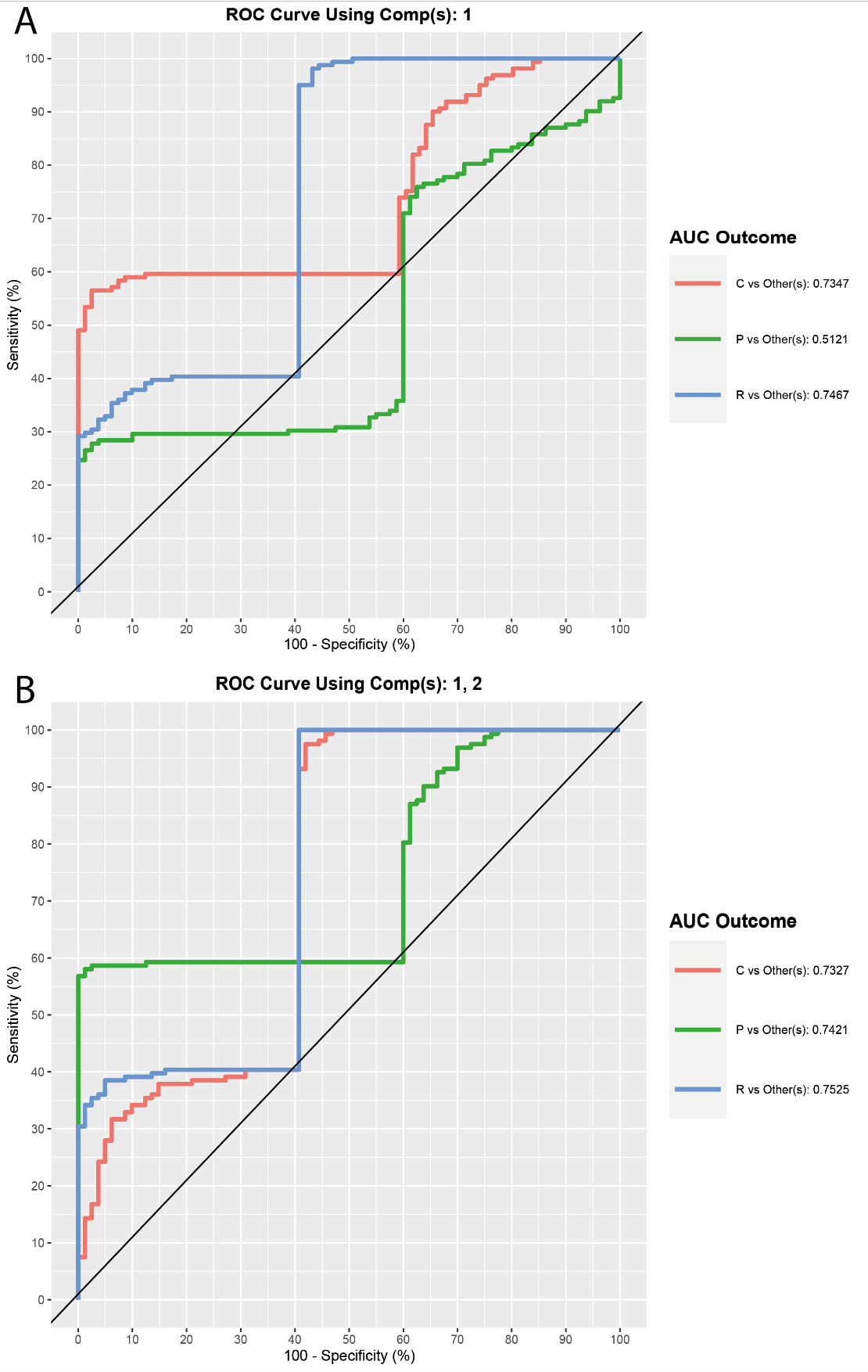


**Figure S8. ROC curve and AUC from the MINT sPLSDA by** **microbial inoculum, Components 1 and 2.** The graphs show a visualisation of classification performance when undergoing the LOGOCV (Leave One Group Out Cross Validation) procedure on the model. The Receiver Operator Curve (ROC) depicts how the Sensitivity (True Positive Rate) and the 100-Specificity (False Positive Rate) change as the classification threshold changes in the MINT sPLSDA by microbial inoculum performed on the chemical features abundance data. Numerical output is the Area Under the Curve (AUC) for each ‘one vs. other’ class comparison that are performed for this component. **A:** The curves show, for component 1, ROC averaged across one-vs-all comparisons.  **B:** The curves show, for component 1 and 2, ROC averaged across one-vs-all comparisons. As PLSDA models are built iteratively (component 2 depends on built component 1), thus it makes sense to plot both components and to look how the error rate decreases when the number of components increases.

**References**

**Rohart F, Eslami A, Matigian N, Bougeard S, Lê Cao K-A**. **2017a**. MINT: a multivariate integrative method to identify reproducible molecular signatures across independent experiments and platforms. *BMC Bioinformatics* **18**: 128.

**Rohart F, Gautier B, Singh A, Cao K-AL**. **2017b**. mixOmics: An R package for ‘omics feature selection and multiple data integration. *PLOS Computational Biology* **13**: e1005752.

**Tada I, Chaleckis R, Tsugawa H, Meister I, Zhang P, Lazarinis N, Dahlén B, Wheelock CE, Arita M**. **2020**. Correlation-Based Deconvolution (CorrDec) To Generate High-Quality MS2 Spectra from Data-Independent Acquisition in Multisample Studies. *Analytical Chemistry* **92**: 11310–11317.

**Tsugawa H, Cajka T, Kind T, Ma Y, Higgins B, Ikeda K, Kanazawa M, VanderGheynst J, Fiehn O, Arita M**. **2015**. MS-DIAL: data-independent MS/MS deconvolution for comprehensive metabolome analysis. *Nature Methods* **12**: 523–526.
